# Estimation of COVID-19 mRNA Vaccine Effectiveness Against Medically Attended COVID-19 in Pregnancy During Periods of Delta and Omicron Variant Predominance in the United States

**DOI:** 10.1001/jamanetworkopen.2022.33273

**Published:** 2022-09-26

**Authors:** Stephanie J. Schrag, Jennifer R. Verani, Brian E. Dixon, Jessica M. Page, Kristen A. Butterfield, Manjusha Gaglani, Gabriela Vazquez-Benitez, Ousseny Zerbo, Karthik Natarajan, Toan C. Ong, Victoria Lazariu, Suchitra Rao, Ryan Beaver, Sascha R. Ellington, Nicola P. Klein, Stephanie A. Irving, Shaun J. Grannis, Salome Kiduko, Michelle A. Barron, John Midturi, Monica Dickerson, Ned Lewis, Melissa S. Stockwell, Edward Stenehjem, William F. Fadel, Ruth Link-Gelles, Kempapura Murthy, Kristin Goddard, Nancy Grisel, Nimish R. Valvi, Bruce Fireman, Julie Arndorfer, Deepika Konatham, Sarah Ball, Mark G. Thompson, Allison L. Naleway

**Affiliations:** 1 CDC COVID-19 Emergency Response Team, Atlanta, Georgia; 2 Center for Biomedical Informatics, Regenstrief Institute, Indianapolis, Indiana; 3 Fairbanks School of Public Health, Indiana University, Indianapolis; 4 Division of Maternal Fetal Medicine, Department of Obstetrics and Gynecology, Intermountain Healthcare, University of Utah, Salt Lake City; 5 Westat, Rockville, Maryland; 6 Baylor Scott & White Health Temple, Texas; 7 Texas A&M University College of Medicine, Temple; 8 HealthPartners Institute, Minneapolis, Minnesota; 9 Kaiser Permanente Vaccine Study Center, Kaiser Permanente Northern California Division of Research, Oakland; 10 Department of Biomedical Informatics, Columbia University Irving Medical Center, New York, New York; 11 NewYork-Presbyterian Hospital, New York; 12 School of Medicine, University of Colorado Anschutz Medical Campus, Aurora; 13 Center for Health Research, Kaiser Permanente Northwest, Portland, Oregon; 14 Indiana University School of Medicine, Indianapolis; 15 Division of Child and Adolescent Health, Department of Pediatrics, Columbia University Vagelos College of Physicians and Surgeons, New York, New York; 16 Department of Population and Family Health, Columbia University Mailman School of Public Health, New York, New York

## Abstract

**Importance:**

Pregnant people are at high risk for severe COVID-19 but were excluded from mRNA vaccine trials; data on COVID-19 vaccine effectiveness (VE) are needed.

**Objective:**

To evaluate the estimated effectiveness of mRNA vaccination against medically attended COVID-19 among pregnant people during Delta and Omicron predominance.

**Design, Setting, and Participants:**

This test-negative, case-control study was conducted from June 2021 to June 2022 in a network of 306 hospitals and 164 emergency department and urgent care (ED/UC) facilities across 10 US states, including 4517 ED/UC encounters and 975 hospitalizations among pregnant people with COVID-19–like illness (CLI) who underwent SARS-CoV-2 molecular testing.

**Exposures:**

Two doses (14-149 and ≥150 days prior) and 3 doses (7-119 and ≥120 days prior) of COVID-19 mRNA vaccine (≥1 dose received during pregnancy) vs unvaccinated.

**Main Outcomes and Measures:**

Estimated VE against laboratory-confirmed COVID-19–associated ED/UC encounter or hospitalization, based on the adjusted odds ratio (aOR) for prior vaccination; VE was calculated as (1 − aOR) × 100%.

**Results:**

Among 4517 eligible CLI-associated ED/UC encounters and 975 hospitalizations, 885 (19.6%) and 334 (34.3%) were SARS-CoV-2 positive, respectively; the median (IQR) patient age was 28 (24-32) years and 31 (26-35) years, 537 (12.0%) and 118 (12.0%) were non-Hispanic Black and 1189 (26.0%) and 240 (25.0%) were Hispanic. During Delta predominance, the estimated VE against COVID-19–associated ED/UC encounters was 84% (95% CI, 69% to 92%) for 2 doses within 14 to 149 days, 75% (95% CI, 5% to 93%) for 2 doses 150 or more days prior, and 81% (95% CI, 30% to 95%) for 3 doses 7 to 119 days prior; estimated VE against COVID-19–associated hospitalization was 99% (95% CI, 96% to 100%), 96% (95% CI, 86% to 99%), and 97% (95% CI, 79% to 100%), respectively. During Omicron predominance, for ED/UC encounters, the estimated VE of 2 doses within 14 to 149 days, 2 doses 150 or more days, 3 doses within 7 to 119 days, and 3 doses 120 or more days prior was 3% (95% CI, −49% to 37%), 42% (95% CI, −16% to 72%), 79% (95% CI, 59% to 89%), and −124% (95% CI, −414% to 2%), respectively; for hospitalization, estimated VE was 86% (95% CI, 41% to 97%), 64% (95% CI, −102% to 93%), 86% (95% CI, 28% to 97%), and −53% (95% CI, −1254% to 83%), respectively.

**Conclusions and Relevance:**

In this study, maternal mRNA COVID-19 vaccination, including booster dose, was associated with protection against medically attended COVID-19. VE estimates were higher against COVID-19–associated hospitalization than ED/UC visits and lower against the Omicron variant than the Delta variant. Protection waned over time, particularly during Omicron predominance.

## Key Points

**Question:**

What is the estimated vaccine effectiveness (VE) of mRNA COVID-19 vaccines against medically attended COVID-19 during pregnancy?

**Findings:**

In a case-control study including 5492 health care encounters sought by pregnant people, the estimated 2-dose VE against COVID-19–associated emergency department and urgent care encounters during Omicron predominance was not significantly different from 0; estimated 3-dose VE (last dose 7-119 days prior) was 79%. Against COVID-19–associated hospitalizations, 2-dose VE (14-149 days prior) was 86%, and 3-dose VE (7-119 days prior) was 86%; 2- and 3-dose VE estimates were not statistically different from 0 for doses 150 and more and 120 and more days prior.

**Meaning:**

In this study, maternal vaccination, including booster dose, was associated with protection against serious COVID-19 during pregnancy.

## Teaser

This case-control study evaluates the estimated effectiveness of mRNA vaccination against medically attended COVID-19 among pregnant people during Delta and Omicron predominance in the United States.

## Introduction

SARS-CoV-2 infection during pregnancy is associated with increased risk of hospitalization, intensive care admission,^1^ preterm delivery, and stillbirth.^2,3^ Although pregnancy was an exclusion criterion in pivotal trials of COVID-19 mRNA vaccines, recommendations for COVID-19 vaccination include pregnant people. Current guidance from the US Centers for Disease Control and Prevention (CDC)^4^ and American College of Obstetricians and Gynecologists^5^ recommends that all pregnant people receive a primary series and booster dose, with a preference for mRNA vaccines. Despite this, COVID-19 vaccine coverage among pregnant people remains low compared with similarly aged nonpregnant individuals.^6^

Evidence of the benefits of COVID-19 vaccination may increase confidence in vaccination among pregnant people. Moreover, because mRNA vaccines are a new class of vaccines with limited human use before the COVID-19 pandemic, postlicensure effectiveness assessments among pregnant people can shed light on whether immune changes associated with pregnancy affect mRNA vaccine performance. Vaccine effectiveness (VE) estimates among pregnant people are accruing,^7,8,9,10,11^ yet, to our knowledge, few studies have focused on more severe COVID-19 outcomes or provided variant-specific estimates. We analyzed data from pregnant people in the VISION Network^12^ to estimate mRNA VE of 2 doses and of a single booster dose against laboratory-confirmed COVID-19–associated emergency department and urgent care (ED/UC) visits and hospitalizations during periods of Delta and Omicron variant predominance.

## Methods

This study followed the Strengthening the Reporting of Observational Studies in Epidemiology (STROBE) reporting guideline and was reviewed and approved by the institutional review boards at participating sites and under a reliance agreement between CDC and the Westat institutional review board. This activity was reviewed by CDC and was conducted consistent with applicable federal law and CDC policy (45 CFR part 46 and 21 CFR part 56). This study presented minimal risk to participants because there was no interaction or intervention with patients; therefore, a waiver of informed consent was granted.

### Data Source and Study Design

VISION Network methods have been previously published.^12^ Briefly, VISION leverages electronic medical record data from 306 hospitals and 164 ED/UC facilities across 10 US states to evaluate COVID-19 VE using a test-negative design. This analysis included hospitalizations or ED/UC visits (referred to together as *medically attended events*) among persons aged 18 to 45 years with a COVID-19–like illness (CLI) diagnosis (based on discharge codes for acute respiratory illness or signs or symptoms of COVID-19) who underwent molecular testing (primarily real-time reverse transcriptase polymerase chain assay) for SARS-CoV-2 during the 14 days before through 72 hours after the medical encounter and who were pregnant at the time of the encounter. Four categories of codes were considered: (1) acute respiratory illness, including respiratory failure, viral or bacterial pneumonia, asthma exacerbation, influenza, and viral illness not otherwise specified; (2) nonrespiratory COVID-19–like illness diagnoses including cause-unspecified gastroenteritis, thrombosis, and acute myocarditis; (3) respiratory signs and symptoms consistent with COVID-19–like illness, including hemoptysis, cough, dyspnea, painful respiration, or hypoxemia; and (4) signs and symptoms of acute febrile illness. One code in any of the 4 categories was sufficient for inclusion. Whether CLI encounters were associated with delivery and pregnancy status at the time of vaccination and CLI encounters was ascertained based on established approaches at each site (eTable 1 in the Supplement). Three health systems used modifications of a published algorithm,^13,14^ 4 used delivery flags from pregnancy data tables based on electronic medical records, and 1 relied on *International Statistical Classification of Diseases and Related Health Problems, Tenth Revision *(*ICD-10*) codes for CLI encounters and surrounding medical encounters. To avoid classifying hospitalizations with incidental SARS-CoV-2 infections among persons screened when admitted for labor and delivery as COVID-19–associated hospitalizations, some relatively nonspecific *ICD* codes for CLI signs and symptoms used in prior VISION publications^12^ and in the ED/UC outcome for this analysis were excluded from the CLI definition for the hospitalization outcome (eTable 2 in the Supplement). A complete list of diagnostic codes included in CLI case definitions for ED/UC visits and hospitalizations for this analysis is presented in eTable 2 in the Supplement. Data on race (Black, White, and other [Asian, American Indian or Alaska Native, Native Hawaiian or other Pacific Islander, multiracial, and other not listed]) and ethnicity (Hispanic and non-Hispanic), which are potential confounders of the association between vaccination and medically attended COVID-19, were obtained from the health systems’ electronic medical record.

The analysis was stratified into 2 periods of predominant variants based on state and local surveillance data: Delta (when the Delta variant accounted for ≥50% of new cases with sequencing data, June 1 to December 15, 2021, depending on site) and Omicron (when the Omicron variant accounted for ≥50% of new cases, December 16, 2021, to February 26, 2022, depending on site) (eTable 3 in the Supplement). Start dates (eTable 3 in the Supplement) varied accordingly and ranged from June 1, 2021, in Colorado to September 11, 2021, in Texas (the earliest date data were available for this site); the end date was June 2, 2022.

To aid in interpretation of the VE results among pregnant people, VE was also estimated for nonpregnant women aged 18 to 45 years in the VISION network for the same time periods and using the same analytic methods. A sensitivity analysis was also conducted excluding participants with any documented SARS-CoV-2 positive result on a molecular test 14 or more days before the current CLI encounter.

### Exposure

Exposures of primary interest were vaccination with 2 doses (14-149 and ≥150 days prior to an eligible CLI event) and 3 doses (7-119 and ≥120 days prior) of mRNA vaccine (either BNT162b2 [Pfizer-BioNTech] or mRNA-1273 [Moderna]), with at least 1 dose administered during the same pregnancy associated with the CLI event. As a secondary analysis, we examined the VE of 2 doses (14-149 and ≥150 days prior) and of 3 doses (7-119 and ≥120 days prior) received any time before the CLI event, including people who only received doses before pregnancy. The following exclusion criteria were applied: recipients of Ad.26.COV2.S (Janssen vaccine [Johnson& Johnson]); recipients of exactly 1 or more than 3 doses of mRNA vaccine; and those for whom fewer than 14 days had elapsed since second dose or fewer than 7 days since third dose.

### Outcome

Outcomes of interest were COVID-19–associated ED/UC CLI visits and COVID-19–associated CLI hospitalizations, defined by a CLI medical encounter (as previously described) with a positive SARS-CoV-2 test result in pregnant persons (and in nonpregnant women aged 18-45 years for reference), during periods of Delta and Omicron variant predominance.

### Statistical Analysis

The association between prior vaccination (most recent dose during pregnancy) and laboratory-confirmed COVID-19 medical encounters (ED/UC visits and hospitalizations) was estimated by comparing the odds of prior vaccination among patients with CLI and positive SARS-CoV-2 results (ie, cases) and those with negative results (ie, controls) using logistic regression, with VE estimated as (1 − adjusted odds ratio [aOR]) × 100%. As in previous VISION analyses, ORs were adjusted for age, geographic region (8 regions, representing the composite of counties with facilities for each health system) (eTable 3 in the Supplement), calendar time (days from January 1, 2021, to the CLI event, based either on preceding test or medical encounter date), and local virus circulation.^12^ Propensity scores were calculated based on the probability of vaccination in test-negative control participants as previously described for VISION,^15^ with the addition of estimated gestational age (included as both a continuous variable and a categorical variable [as estimated trimester of pregnancy]) because of its potential to confound the association between vaccination and SARS-CoV-2 infection among pregnant people. Factors unbalanced after estimation of propensity scores were included as covariates in the models. The referent group for all VE analyses was 0 doses (unvaccinated individuals). All analyses were stratified by period of predominant SARS-CoV-2 variant. VE estimates were not calculated for strata that included fewer than 20 encounters with prior vaccination.

A standard mean or proportion difference of 0.20 or greater indicated a nonnegligible difference in distributions of characteristics by vaccination or infection status. When calculating SMDs for differences in characteristics across COVID-19 vaccination status, we calculated SMD as the average of the 3 absolute values of the SMD for unvaccinated vs each vaccination status category individually (unvaccinated vs 2 doses 14-149 days earlier, unvaccinated vs 2-dose ≥150 days earlier, and unvaccinated vs 3 mRNA doses ≥7 days earlier). The average SMD calculation comparing negative SARS-CoV-2 test result and positive SARS-CoV-2 test result was generated by directly calculating the SMD for negative SARS-CoV-2 test result and positive SARS-CoV-2 test result.

For VE estimates, 2-sided 95% CIs were calculated for each VE point estimate, and nonoverlapping intervals were considered to be significantly different. Analyses were conducted using R version 4.1.2 (R Project for Statistical Computing).

## Results

Among 5492 encounters, there were 4517 eligible CLI ED/UC visits among pregnant persons (eFigure 1 in the Supplement); 885 (19.6%) had a positive SARS-CoV-2 test result; the median age among participants was 28 (24-32) years (Table 1); 537 (11.9%) were non-Hispanic Black, 1189 (26.3%) Hispanic, and 2286 (50.6%) non-Hispanic White. The timing of ED/UC visits during pregnancy was 1218 (27.0%) first trimester, 1647 (36.5%) second trimester, and 1652 (36.6%) third trimester. A total of 2861 visits (63.3%) occurred during Delta variant predominance and 1656 (36.7%) during Omicron variant predominance. Among pregnant persons with a CLI ED/UC visit, 3380 (74.8%) were unvaccinated; among those vaccinated with at least 1 dose during pregnancy, 721 (16.0%) had received 2 doses of mRNA vaccine, including 523 (11.6%) with second dose 14 to 149 days prior and 198 (4.4%) with second dose 150 or more days prior; 416 (9.2%) had received 3 doses, including 351 (7.8%) with third dose 7 to 119 days prior and 65 (1.4%) with third dose 120 or more days prior. Among those vaccinated with documented product information, 769 (67.6%) received BNT162b2 and 321 (28.2%) mRNA-1273. Vaccination was initiated before pregnancy in 589 individuals (51.8%); 341 (30.0%), 192 (16.9%), and 15 (1.3%) received their first dose of mRNA vaccine during the first, second, and third trimesters of pregnancy, respectively.

**Table 1.** Characteristics of Emergency Department and Urgent Care Encounters Among Pregnant People With COVID-19–Like Illness by COVID-19 mRNA Vaccination Status and SARS-CoV-2 Test Result, 10 States, June 1, 2021, to June 2, 2022^a^

Characteristic	Total, No. (column %) (N = 4517)	mRNA COVID-19 vaccination status, No. (row %)						SMD^c^	Positive SARS-CoV-2 test result, No. (row %) (n = 885)	SMD^c^
		Unvaccinated (n = 3380)	2 doses^b^		3 doses^b^					
			14-149 d earlier (n = 523)	≥150 d earlier (n = 198)	7-119 d earlier (n = 351)		≥120 d earlier (n = 65)			
Pregnancy trimester of CLI event										
First	1218 (27.0)	1095 (89.9)	64 (5.3)	0	59 (4.8)		0	0.82	217 (17.8)	0.08
Second	1647 (36.5)	1208 (73.3)	235 (14.3)	31 (1.9)	150 (9.1)		23 (1.4)		345 (20.9)	
Third	1652 (36.6)	1077 (65.2)	224 (13.6)	167 (10.1)	142 (8.6)		42 (2.5)		323 (19.6)	
CLI event associated with delivery										
No	4175 (92.4)	3107 (74.4)	497 (11.9)	180 (4.3)	329 (7.9)		62 (1.5)	0.09	810 (19.4)	0.04
Yes	342 (7.6)	273 (79.8)	26 (7.6)	18 (5.3)	22 (6.4)		3 (0.9)		75 (21.9)	
Variant predominance period										
B.1.617.2 (Delta)	2861 (63.3)	2282 (79.8)	377 (13.2)	121 (4.2)	79 (2.8)		2 (0.1)	0.77	464 (16.2)	0.28
B.1.1.529 (Omicron)	1656 (36.7)	1098 (66.3)	146 (8.8)	77 (4.6)	272 (16.4)		63 (3.8)		421 (25.4)	
Sites										
Baylor Scott & White Health	90 (2.0)	73 (81.1)	13 (14.4)	1 (1.1)	2 (2.2)		1 (1.1)	0.55	34 (37.8)	0.34
Columbia University	271 (6.0)	192 (70.8)	49 (18.1)	16 (5.9)	13 (4.8)		1 (0.4)		34 (12.5)	
HealthPartners	301 (6.7)	183 (60.8)	47 (15.6)	17 (5.6)	39 (13.0)		15 (5.0)		68 (22.6)	
Intermountain Healthcare	1593 (35.3)	1213 (76.1)	183 (11.5)	70 (4.4)	107 (6.7)		20 (1.3)		231 (14.5)	
Kaiser Permanente Northern California	961 (21.3)	636 (66.2)	146 (15.2)	50 (5.2)	113 (11.8)		16 (1.7)		197 (20.5)	
Kaiser Permanente Northwest	318 (7.0)	223 (70.1)	30 (9.4)	13 (4.1)	45 (14.2)		7 (2.2)		96 (30.2)	
Regenstrief Institute	617 (13.7)	550 (89.1)	35 (5.7)	14 (2.3)	15 (2.4)		3 (0.5)		138 (22.4)	
University of Colorado	366 (8.1)	310 (84.7)	20 (5.5)	17 (4.6)	17 (4.6)		2 (0.5)		87 (23.8)	
Age, y										
18-24	1322 (29.3)	1139 (86.2)	113 (8.5)	36 (2.7)	28 (2.1)		6 (0.5)	0.54	244 (18.5)	0.05
25-34	2515 (55.7)	1833 (72.9)	301 (12.0)	128 (5.1)	214 (8.5)		39 (1.6)		509 (20.2)	
35-55	680 (15.1)	408 (60.0)	109 (16.0)	34 (5.0)	109 (16.0)		20 (2.9)		132 (19.4)	
Race and ethnicity										
Hispanic	1189 (26.3)	869 (73.1)	171 (14.4)	57 (4.8)	84 (7.1)		8 (0.7)	0.46	223 (18.8)	0.12
Non-Hispanic										
Black	537 (11.9)	464 (86.4)	46 (8.6)	9 (1.7)	16 (3.0)		2 (0.4)		135 (25.1)	
Other^d^	362 (8.0)	220 (60.8)	50 (13.8)	27 (7.5)	54 (14.9)		11 (3.0)		69 (19.1)	
White	2286 (50.6)	1702 (74.5)	247 (10.8)	102 (4.5)	191 (8.4)		44 (1.9)		431 (18.9)	
Unknown	143 (3.2)	125 (87.4)	9 (6.3)	3 (2.1)	6 (4.2)		0		27 (18.9)	
Chronic respiratory condition^e^										
No	4182 (92.6)	3116 (74.5)	494 (11.8)	187 (4.5)	324 (7.7)		61 (1.5)	0.06	812 (19.4)	0.04
Yes	335 (7.4)	264 (78.8)	29 (8.7)	11 (3.3)	27 (8.1)		4 (1.2)		73 (21.8)	
Chronic nonrespiratory condition^f^										
No	4155 (92.0)	3098 (74.6)	489 (11.8)	183 (4.4)	326 (7.8)		59 (1.4)	0.04	812 (19.5)	0.01
Yes	362 (8.0)	282 (77.9)	34 (9.4)	15 (4.1)	25 (6.9)		6 (1.7)		73 (20.2)	
ICU										
No	4490 (99.4)	3356 (74.7)	523 (11.6)	198 (4.4)	349 (7.8)		64 (1.4)	0.08	877 (19.5)	0.05
Yes	27 (0.6)	24 (88.9)	0	0	2 (7.4)		1 (3.7)		8 (29.6)	
Immunocompromised status										
No	4495 (99.5)	3362 (74.8)	521 (11.6)	197 (4.4)	350 (7.8)		65 (1.4)	0.04	882 (19.6)	0.03
Yes	22 (0.5)	18 (81.8)	2 (9.1)	1 (4.5)	1 (4.5)		0		3 (13.6)	
History of SARS-CoV-2 infection										
No	3934 (87.1)	2940 (74.7)	457 (11.6)	175 (4.4)	305 (7.8)		57 (1.4)	0.02	828 (21.0)	0.27
Yes	583 (12.9)	440 (75.5)	66 (11.3)	23 (3.9)	46 (7.9)		8 (1.4)		57 (9.8)	
Vaccine product, No./total No. (%)										
Combination of mRNA products	47/1137 (4.1)	NA	0	0	38/47 (80.9)		9/47 (19.1)	NA	2/47 (4.3)	0.31
Moderna	321/1137 (28.2)	NA	142/321 (44.2)	59/321 (18.4)	100/321 (31.2)		20/321 (6.2)		22/321 (6.9)	
Pfizer-BioNTech	769/1137 (67.6)	NA	381/769 (49.5)	139/769 (18.1)	213/769 (27.7)		36/769 (4.7)		93/769 (12.1)	
Timing of first dose, No./total No. (%)										
Before current pregnancy	589/1137 (51.8)	NA	97/589 (16.5)	87/589 (14.8)	340/589 (57.7)		65/589 (11.0)	NA	60/589 (10.2)	0.19
First trimester	341/1137 (30.0)	NA	224/341 (65.7)	106/341 (31.1)	11/341 (3.2)		0		34/341 (10.0)	
Second trimester	192/1137 (16.9)	NA	187/192 (97.4)	5/192 (2.6)	0		0		23/192 (12.0)	
Third trimester	15/1137 (1.3)	NA	15/15 (100.0)	0	0		0		0	
Timing of second dose, No./total No. (%)										
Before current pregnancy	395/1137 (34.7)	NA	0	0	330/395 (83.5)		65/395 (16.5)	NA	35/395 (8.9)	0.26
First trimester	465/1137 (40.9)	NA	263/465 (56.6)	181/465 (38.9)	21/465 (4.5)		0		55/465 (11.8)	
Second trimester	234/1137 (20.6)	NA	217/234 (92.7)	17/234 (7.3)	0		0		26/234 (11.1)	
Third trimester	43/1137 (3.8)	NA	43/43 (100.0)	0	0		0		1/43 (2.3)	
Timing of third dose, No./total No. (%)										
First trimester	180/416 (43.3)	NA	0	0	157/180 (87.2)		23/180 (12.8)	NA	18/180 (10.0)	0.15
Second trimester	186/416 (44.7)	NA	0	0	179/186 (96.2)		7/186 (3.8)		14/186 (7.5)	
Third trimester	50/416 (12)	NA	0	0	50/50 (100.0)		0		4/50 (8.0)	
2 doses during current pregnancy, No./total No. (%)										
No	184/721 (25.5)	NA	97/184 (52.7)	87/184 (47.3)	0		0	NA	24/184 (13.0)	0.10
Yes	537/721 (74.5)	NA	426/537 (79.3)	111/537 (20.7)	0		0		57/537 (10.6)	
Timing of doses										
First dose, before current pregnancy										
Second dose, first trimester	182/721 (25.2)	NA	95/182 (52.2)	87/182 (47.8)	NA	NA		NA	24/182 (13.2)	NA
Second dose, second trimester	2/721 (0.3)	NA	2/2 (100.0)	0	NA	NA			0	
First dose, first trimester										
Second dose, first trimester	262/721 (36.3)	NA	168/262 (64.1)	94/262 (35.9)	NA	NA		NA	30/262 (11.5)	NA
Second dose, second trimester	66/721 (9.2)	NA	54/66 (81.8)	12/66 (18.2)	NA	NA			4/66 (6.0)	
Second dose, third trimester	2/721 (0.3)	NA	2/2 (100.0)	0	NA	NA			0	
First dose, second trimester										
Second dose, second trimester	166/721 (23.0)	NA	161/166 (97.0)	5/166 (3.0)	NA	NA		NA	22/166 (13.3)	NA
Second dose, third trimester	26/721 (3.6)	NA	26/26 (100.0)	0	NA	NA			1/26 (3.8)	
First and second dose, third trimester	15/721 (2.1)	NA	15 (100.0)	0	NA	NA			0	
3 doses during current pregnancy, No./total No. (%)										
No	405 (97.4)	NA	NA	NA	375 (92.6)		30 (7.4)	NA	36 (8.9)	0.24
Yes	11 (2.6)	NA	NA	NA	11 (100.0)		0		0	
Timing of doses										
First dose before current pregnancy										
Second dose before current pregnancy										
Third dose, first trimester	180/405 (4.0)	NA	NA	NA	157/180 (87.2)		23/180 (12.8)	NA	18/180 (10.0)	NA
Third dose, second trimester	185/405 (4.1)	NA	NA	NA	178/185 (96.2)		7/185 (3.8)		14/185 (7.6)	
Third dose, third trimester	30/405 (7.4)	NA	NA	NA	30/30 (100.0)		0		0	
Second dose, first trimester										
Third dose, second trimester	1/405 (0.2)	NA	NA	NA	1/1 (100.0)		0	NA	0	NA
Third dose, third trimester	9/405 (2.2)	NA	NA	NA	9/9 (100.0)		0		3/9 (33.3)	
First and second dose, first trimester; third dose third trimester	11/405 (2.7)	NA	NA	NA	11/11 (100.0)		0	NA	1/11 (9.1)	NA

Abbreviations: CLI, COVID-19–like illness; ICU, intensive care unit; SMD, standardized mean or proportion difference.

^a^ Information on how CLI was classified and the data sources is available in the Methods section.

^b^ Vaccination was defined as having received the listed number of doses of COVID-19 BNT162b2 (Pfizer-BioNTech) or mRNA-1273 (Moderna) vaccine 14 or more days (for 2 doses) or 7 or more days (for 3 doses) before the medical event index date, which was the date of respiratory specimen collection associated with the most recent positive or negative SARS-CoV-2 test result before medical event or the admission date if testing only occurred after the admission. Among those with 3 doses, 3 during Delta and 1 during Omicron received the third dose fewer than 150 days after their second dose and none of these 4 had documented immunocompromised status. In this analysis, all vaccinated individuals received at least their most recent dose during the pregnancy where the CLI event occurred.

^c^ An absolute SMD of 0.20 or greater indicates a nonnegligible difference in the distribution of characteristics for vaccinated categories vs unvaccinated patients and for positive vs negative test results. All SMDs are reported as the absolute SMD. More information on calculating SMDs is available in the Methods section.

^d^ Unknown race and ethnicity includes American Indian or Alaska Native, Asian, Native Hawaiian or other Pacific Islander, other not listed, and multiple races.

^e^ Chronic respiratory condition was defined as the presence of discharge code for asthma, chronic obstructive pulmonary disease, or other lung disease using diagnosis codes from the *International Classification of Diseases, Ninth Revision* and *International Statistical Classification of Diseases and Related Health Problems, Tenth Revision*.

^f^ Chronic nonrespiratory condition was defined as the presence of discharge code for heart failure, ischemic heart disease, hypertension, other heart disease, stroke, other cerebrovascular disease, diabetes type 1 or 2, other diabetes, metabolic disease, clinical obesity, clinically underweight, kidney disease, liver disease, blood disorder, immunosuppression, organ transplantation, cancer, neurological disorder, musculoskeletal disorder, Down Syndrome, and dementia.

**Figure 1.**
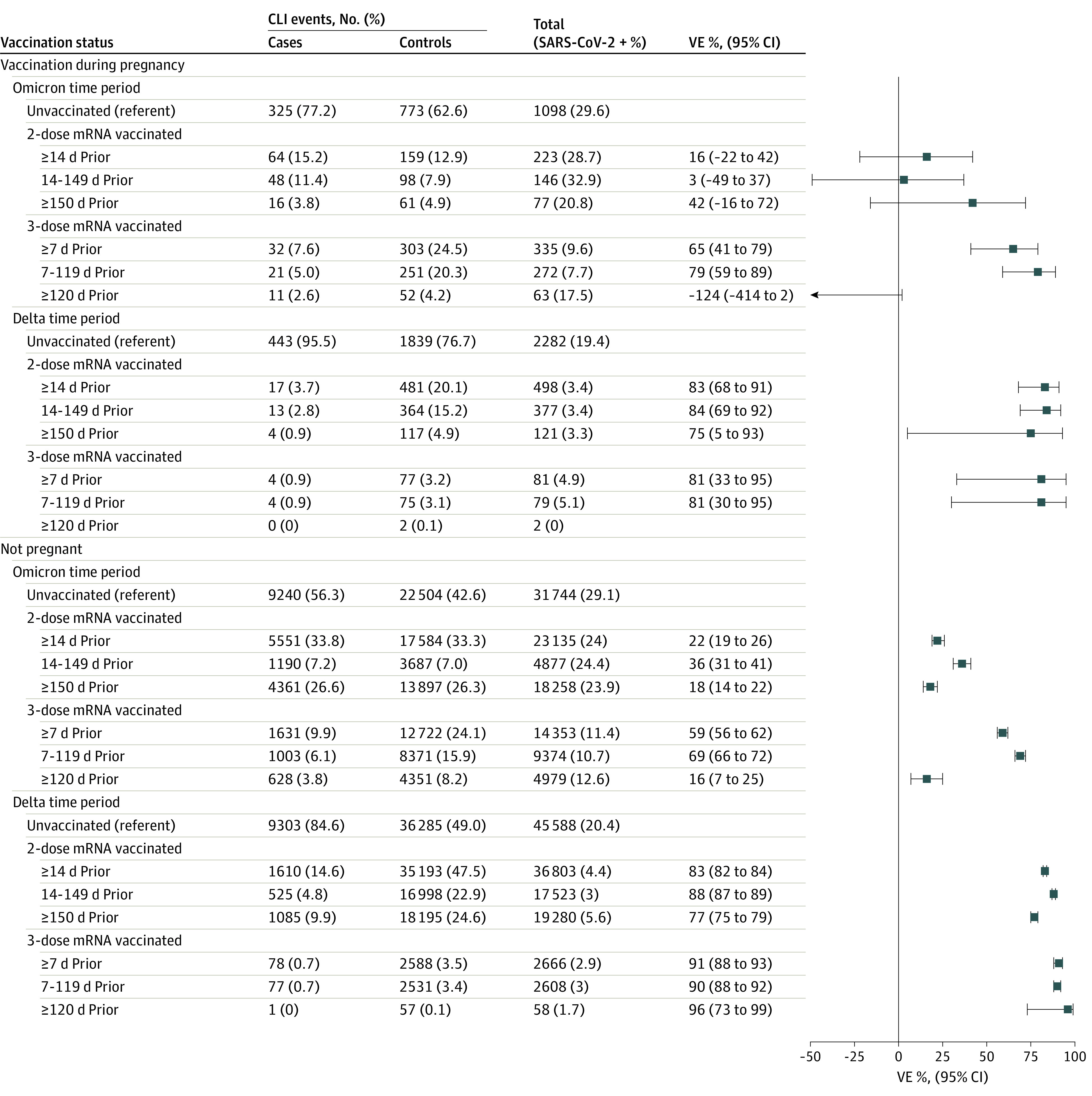
mRNA COVID-19 Vaccine Effectiveness (VE) Against Laboratory-Confirmed COVID-19–Associated Emergency Department and Urgent Care Encounters Among Pregnant People and Nonpregnant Women, VISION Network, 10 States, June 1, 2021, to June 2, 2022 The VISION Network includes Baylor Scott & White Health (Texas), Columbia University Irving Medical Center (New York), HealthPartners (Minnesota and Wisconsin), Intermountain Healthcare (Utah), Kaiser Permanente Northern California (California), Kaiser Permanente Northwest (Oregon and Washington), Regenstrief Institute (Indiana), and University of Colorado (Colorado). Vaccinated pregnant people included in this analysis received at least their most recent dose during pregnancy; among those with 3 doses, 3 participants during Delta and 1 during Omicron received dose 3 less than 150 days after dose 2, and none of these 4 had documented immunocompromised status. Among vaccinated nonpregnant people with 3 doses, 273 in the Delta period and 324 in the Omicron period received the third dose less than 150 days after the second dose. VE was calculated as described in the Methods section. VE could not be calculated for vaccination during pregnancy during the Delta period among individuals who received 3 doses of mRNA vaccine 120 or more days because the stratum included fewer than 20 encounters with prior vaccination. CLI indicates COVID-19–like illness.

During Delta variant predominance, the estimated VE of 2 mRNA vaccine doses 14 to 149 days prior against COVID-19–associated ED/UC visits was 84% (95% CI, 69%-92%) (Figure 1); for 2 doses received 150 or more days prior, it was 75% (95% CI, 5%-93%). The estimated VE of 3 doses 7 to 119 days prior was 81% (95% CI, 30%-95%); the sample was not sufficient to estimate VE for 2 doses 120 or more days prior. During Omicron variant predominance, the estimated VEs against COVID-19–associated ED/UC visits were 3% (95% CI, −49% to 37%) for 2 doses within 14 and 149 days and 42% (95% CI, −16% to 72%) for 2 doses 120 or more day prior. The estimated VE of 3 doses 7 to 119 days prior was 79% (95% CI, 59%-89%); the VE for 3 doses 120 or more days prior was −124% (95% CI, −414% to 2%).

Among 975 eligible hospitalizations among pregnant persons, 334 (34.3%) had a positive SARS-CoV-2 result; the median (IQR) age among participants was 31 (26-35) years (Table 2); 118 (12.1%) were non-Hispanic Black, 240 (24.5%) Hispanic, and 440 (45.1%) non-Hispanic White. The timing of hospitalizations during pregnancy was 31 (3.2%) in the first trimester, 161 (16.5%) second trimester, and 783 (80.3%) third trimester. Two-thirds of CLI hospitalizations (638 of 975 [65.4%]) (Table 2) were associated with delivery. CLI hospitalizations among pregnant people who delivered, who did not deliver, and among nonpregnant women were similar in terms of severity characteristics (Table 3). Among CLI hospitalizations associated with delivery, the frequency of preterm delivery (<37 weeks gestation) was 38.3% (245 of 638) overall and 54.9% (90of 164) among those who were SARS-CoV-2 positive. A total of 679 hospitalizations (69.6%) occurred during Delta variant predominance and 337 (34.6%) during Omicron variant predominance. Among pregnant persons with a CLI hospitalization, 670 (68.7%) were unvaccinated; among those vaccinated with at least 1 dose during pregnancy, 199 (20.4%) had received 2 doses of mRNA vaccine, including 121 (12.4%) with second dose 14 to 149 days before and 78 (8.0%) with second dose 150 or more days before; 106 (10.9%) had received 3 doses, including 82 (8.4%) with third dose 7 to 119 days before and 24 (2.5%) with second dose 120 days or more before. Among the vaccinated with documented product information, 187 (61.3%) received BNT162b2 and 111 (36.4%) mRNA-1273. Vaccination was initiated prior to pregnancy in 118 individuals (38.7%) and in the first, second, and third trimesters of pregnancy among 84 (27.5%), 84 (27.6%), and 19 (6.2%), respectively.

**Table 2.** Characteristics of Hospitalizations Among Pregnant People With CLI by COVID-19 mRNA Vaccination Status and SARS-CoV-2 Test Result, 10 States, June 1, 2021, to June 2, 2022^a^

Characteristic	Total, No. (column %) (N = 975)	mRNA COVID-19 vaccination status, No. (row %)					SMD^c^	Positive SARS-CoV-2 test result, No. (row %)	SMD^c^
		Unvaccinated (n = 670)	2 doses^b^		3 doses^b^				
			14-149 d earlier	≥150 d earlier	7-119 d earlier	≥120 d earlier			
Pregnancy trimester of CLI event									
First	31 (3.2)	30 (96.8)	0	0	1 (3.2)	0	0.47	17 (54.8)	0.29
Second	161 (16.5)	135 (83.9)	12 (7.5)	5 (3.1)	7 (4.3)	2 (1.2)		75 (46.6)	
Third	783 (80.3)	505 (64.5)	109 (13.9)	73 (9.3)	74 (9.5)	22 (2.8)		242 (30.9)	
CLI event associated with delivery									
No	337 (34.6)	263 (78.0)	32 (9.5)	12 (3.6)	20 (5.9)	10 (3.0)	0.30	170 (50.4)	0.53
Yes	638 (65.4)	407 (63.8)	89 (13.9)	66 (10.3)	62 (9.7)	14 (2.2)		164 (25.7)	
Variant predominance period									
B.1.617.2 (Delta)	679 (69.6)	498 (73.3)	101 (14.9)	57 (8.4)	23 (3.4)	0	0.93	258 (38.0)	0.26
B.1.1.529 (Omicron)	296 (30.4)	172 (58.1)	20 (6.8)	21 (7.1)	59 (19.9)	24 (8.1)		76 (25.7)	
Sites									
Baylor Scott & White Health	34 (3.5)	27 (79.4)	5 (14.7)	1 (2.9)	1 (2.9)	0	0.73	8 (23.5)	0.41
Columbia University	81 (8.3)	50 (61.7)	13 (16.0)	13 (16.0)	4 (4.9)	1 (1.2)		16 (19.8)	
HealthPartners	17 (1.7)	11 (64.7)	1 (5.9)	2 (11.8)	2 (11.8)	1 (5.9)		3 (17.6)	
Intermountain Healthcare	105 (10.8)	83 (79.0)	7 (6.7)	9 (8.6)	5 (4.8)	1 (1.0)		27 (25.7)	
Kaiser Permanente Northern California	337 (34.6)	192 (57.0)	53 (15.7)	34 (10.1)	46 (13.6)	12 (3.6)		131 (38.9)	
Kaiser Permanente Northwest	68 (7.0)	38 (55.9)	9 (13.2)	5 (7.4)	11 (16.2)	5 (7.4)		20 (29.4)	
Regenstrief Institute	176 (18.1)	147 (83.5)	16 (9.1)	2 (1.1)	8 (4.5)	3 (1.7)		84 (47.7)	
University of Colorado	157 (16.1)	122 (77.7)	17 (10.8)	12 (7.6)	5 (3.2)	1 (0.6)		45 (28.7)	
Age, y									
18-24	169 (17.3)	143 (84.6)	18 (10.7)	5 (3.0)	3 (1.8)	0	0.63	53 (31.4)	0.29
25-34	528 (54.2)	379 (71.8)	62 (11.7)	40 (7.6)	35 (6.6)	12 (2.3)		191 (36.2)	
35-55	278 (28.5)	148 (53.2)	41 (14.7)	33 (11.9)	44 (15.8)	12 (4.3)		90 (32.4)	
Race and ethnicity									
Hispanic	240 (24.6)	167 (69.6)	31 (12.9)	23 (9.6)	15 (6.2)	4 (1.7)	0.42	78 (32.5)	0.25
Non-Hispanic									
Black	118 (12.1)	97 (82.2)	9 (7.6)	5 (4.2)	7 (5.9)	0		59 (50.0)	
Other^d^	141 (14.6)	80 (56.7)	22 (15.6)	14 (9.9)	20 (14.2)	5 (3.5)		46 (32.6)	
White	440 (45.1)	305 (69.3)	53 (12.0)	33 (7.5)	35 (8.0)	14 (3.2)		140 (31.8)	
Unknown	36 (3.7)	21 (58.3)	6 (16.7)	3 (8.3)	5 (13.9)	1 (2.8)		11 (30.6)	
Chronic respiratory condition^e^									
No	513 (52.6)	316 (61.6)	78 (15.2)	51 (9.9)	53 (10.3)	15 (2.9)	0.35	128 (25.0)	0.45
Yes	462 (47.4)	354 (76.6)	43 (9.3)	27 (5.8)	29 (6.3)	9 (1.9)		206 (44.6)	
Chronic non respiratory condition^f^									
No	447 (45.8)	308 (68.9)	52 (11.6)	42 (9.4)	34 (7.6)	11 (2.5)	0.08	126 (28.2)	0.25
Yes	528 (54.2)	362 (68.6)	69 (13.1)	36 (6.8)	48 (9.1)	13 (2.5)		208 (39.4)	
ICU									
No	853 (87.5)	574 (67.3)	109 (12.8)	71 (8.3)	76 (8.9)	23 (2.7)	0.22	275 (32.2)	0.23
Yes	122 (12.5)	96 (78.7)	12 (9.8)	7 (5.7)	6 (4.9)	1 (0.8)		59 (48.4)	
Immunocompromised status									
No	940 (96.4)	648 (68.9)	115 (12.2)	75 (8.0)	78 (8.3)	24 (2.6)	0.11	326 (34.7)	0.10
Yes	35 (3.6)	22 (62.9)	6 (17.1)	3 (8.6)	4 (11.4)	0		8 (22.9)	
History of SARS-CoV-2 infection									
No	888 (91.1)	612 (68.9)	110 (12.4)	70 (7.9)	74 (8.3)	22 (2.5)	0.03	329 (37.0)	0.45
Yes	87 (8.9)	58 (66.7)	11 (12.6)	8 (9.2)	8 (9.2)	2 (2.3)		5 (5.7)	
Vaccine product, No./total No. (%)							NA		0.22
Combination of mRNA products	7/305 (2.3)	NA	0	0	7/7 (100.0)	0		1/7 (14.3)	
mRNA-1273	111/305 (36.4)	NA	44/111 (39.6)	33/111 (29.7)	33/111 (29.7)	1/111 (0.9)		6/111 (5.4)	
BNT162b2	187/305 (61.3)	NA	77/187 (41.2)	45/187 (24.1)	58/187 (31.0)	7/111 (3.7)		14/111 (7.5)	
Timing of first dose, No./total No. (%)									
Before current pregnancy	118/305 (38.7)	NA	2/118 (1.7)	23/118 (19.5)	85/118 (72.0)	8/118 (6.8)	NA	11/118 (9.3)	0.56
First trimester	84/305 (27.5)	NA	22/84 (26.2)	49/84 (58.3)	13/84 (15.5)	0		7/84 (8.3)	
Second trimester	84/305 (27.5)	NA	78/84 (92.9)	6/84 (7.1)	0	0		3/84 (3.6)	
Third trimester	19/305 (6.2)	NA	19/19 (100.0)	0	0	0		0	
Timing of second dose, No./total No. (%)									
Before current pregnancy	84/305 (27.5)	NA	0	0	76/84 (90.5)	8/84 (9.5)	NA	8/84 (9.5)	0.62
First trimester	90/305 (29.5)	NA	9/90 (10.0)	59/90 (65.6)	22/90 (24.4)	0		8/90 (8.9)	
Second trimester	94/305(30.8)	NA	75/94 (79.8)	19/94 (20.2)	0	0		5/94 (5.3)	
Third trimester	37/305 (12.1)	NA	37/37 (100.0)	0	0	0		0	
Timing of third dose									
First trimester	10/106 (9.4)	NA	0	0	5/10 (50.0)	5/10 (50.0)	NA	2/10 (20.0)	0.40
Second trimester	52/106 (49.1)	NA	0	0	49/52 (94.2)	3/52 (5.8)		4/52 (7.7)	
Third trimester	44/106 (41.5)	NA	0	0	44/44 (100.0)	0		3/44 (6.8)	
2 doses during current pregnancy, No./total No. (%)									
No	25/199 (12.6)	NA	2/25 (8.0)	23/25 (92.0)	0	0	NA	2/25 (8.0)	0.12
Yes	174/199 (87.4)	NA	119/199 (68.4)	55/199 (31.6)	0	0		10/199 (5.7)	
Timing of doses									
First dose before current pregnancy									
Second dose, first trimester	24/199 (12.1)	NA	1/24 (4.2)	23/24 (95.8)	NA	NA	NA	2/24 (8.3)	NA
Second dose, second trimester	1/199 (0.5)	NA	1/1 (100.0)	0	NA	NA		0	
First dose, first trimester									
Second dose, first trimester	44/199 (22.1)	NA	8/44 (18.2)	36/44 (81.8)	NA	NA	NA	5/44 (11.4)	NA
Second dose, second trimester	26/199 (13.1)	NA	13/26 (50.0)	13/26 (50.0)	NA	NA		2/26 (7.7)	
Second dose. third trimester	1/199 (0.5)	NA	1/1 (100.0)	0	NA	NA		0	
First dose, second trimester									
Second dose, second trimester	67/199 (33.7)		61/67 (91.0)	6/67 (9.0)	NA	NA	NA	3/67 (4.5)	NA
First dose, third trimester	17/199 (8.5)		17/17 (100.0)	0	NA	NA		0	
First and second doses, third trimester	19/199 (9.5)		19/19 (100.0)	0	NA	NA	NA	0	NA
3 doses during current pregnancy, No./total No. (%)									
No	93/106 (87.7)	0	0	0	85/93 (91.4)	8/93 (8.6)	NA	9/93 (9.7)	0.56
Yes	13/106 (12.3)	0	0	0	13/13 (100.0)	0		0	
Timing of doses									
First dose before current pregnancy									
Second dose, before current pregnancy									
Third dose, first trimester	10/106 (9.4)	NA	NA	NA	5/10 (50.0)	5/10 (50.0)	NA	2/10 (20.0)	NA
Third dose, second trimester	50/106 (47.2)	NA	NA	NA	47/50 (94.0)	3/50 (6.0)		4/50 (8.0)	
Third dose, third trimester	24/106 (22.6)	NA	NA	NA	24/24 (100.0)	0		2/24 (8.3)	
Second dose, first trimester									
Third dose, second trimester	1/106 (0.9)	NA	NA	NA	1/1 (100.0)	0	NA	0	NA
Third dose, third trimester	8/106 (7.5)	NA	NA	NA	8/8 (100.0)	0		1/8 (12.5)	
First and second doses, first trimester									
Third dose, second trimester	1/106 (0.9)	NA	NA	NA	1/1 (100.0)	0	NA	0	NA
Third dose, third trimester	12/106 (11.3)	NA	NA	NA	12/12 (100.0)	0		0	

Abbreviations: CLI, COVID-19–like illness; ICU, intensive care unit; SMD, standardized mean or proportion difference.

^a^ Information on how CLI was classified and the data sources is available in the Methods section.

^b^ Vaccination was defined as having received the listed number of doses of COVID-19 BNT162b2 (Pfizer-BioNTech) or mRNA-1273 (Moderna) vaccine 14 or more days (for 2 doses) or 7 or more days (for 3 doses) before the medical event index date, which was the date of respiratory specimen collection associated with the most recent positive or negative SARS-CoV-2 test result before medical event or the admission date if testing only occurred after the admission. Among those with 3 doses, 3 during Delta and 1 during Omicron received the third dose fewer than 150 days after their second dose and none of these 4 had documented immunocompromised status. In this analysis, all vaccinated individuals received at least their most recent dose during the pregnancy where the CLI event occurred.

^c^ An absolute SMD of 0.20 or greater indicates a nonnegligible difference in the distribution of characteristics for vaccinated categories vs unvaccinated patients and for positive vs negative test results. All SMDs are reported as the absolute SMD. More information on calculating SMDs is available in the Methods section.

^d^ Unknown race and ethnicity includes American Indian or Alaska Native, Asian, Native Hawaiian or other Pacific Islander, other not listed, and multiple races.

^e^ Chronic respiratory condition was defined as the presence of discharge code for asthma, chronic obstructive pulmonary disease, or other lung disease using diagnosis codes from the *International Classification of Diseases, Ninth Revision* and *International Statistical Classification of Diseases and Related Health Problems, Tenth Revision*.

^f^ Chronic nonrespiratory condition was defined as the presence of discharge code for heart failure, ischemic heart disease, hypertension, other heart disease, stroke, other cerebrovascular disease, diabetes type 1 or 2, other diabetes, metabolic disease, clinical obesity, clinically underweight, renal disease, liver disease, blood disorder, immunosuppression, organ transplantation, cancer, neurological disorder, musculoskeletal disorder, Down Syndrome, and dementia.

**Table 3.** Severity Indicators During Hospitalization Among Pregnant and Nonpregnant People With CLI by SARS-CoV-2 Test Result, 10 States, June 1, 2021, to June 2, 2022^a^^,^^b^

Indicator	No. (%)											
	All CLI hospitalizations among pregnant people (including pregnant at discharge and delivery during hospitalization)			CLI hospitalizations among pregnant people still pregnant at discharge (no delivery during CLI hospitalization)^c^			CLI hospitalizations among pregnant people with delivery during hospitalization^d^			CLI hospitalizations among nonpregnant women 18-45 y old		
	Total medical events (n = 975)	SARS-CoV-2		Total medical events (n = 338)	SARS-CoV-2		Total medical events (n = 637)	SARS-CoV-2		Total medical events (n = 9297)	SARS-CoV-2	
		Negative (n = 641)	Positive (n = 336)		Negative (n = 168)	Positive (n = 170)		Negative (n = 473)	Positive (n = 164(		Negative (n = 6448)	Positive (n = 2849)
Age group, y												
18-24	169 (17.3)	116 (18.1)	53 (15.8)	59 (17.5)	37 (22.0)	22 (12.9)	110 (17.3)	79 (16.7)	31 (18.9)	1264 (13.6)	1007 (15.6)	257 (9.0)
25-34	528 (54.2)	337 (52.6)	191 (56.8)	189 (55.9)	80 (47.6)	109 (64.1)	339 (53.2)	257 (54.3)	82 (50.0)	2883 (31.0)	2002 (31.0)	881 (30.9)
35-45	278 (28.5)	188 (29.3)	90 (26.8)	90 (26.6)	51 (30.4)	39 (22.9)	187 (29.4)	136 (28.8)	51 (31.1)	5150 (55.4)	3439 (53.3)	1711 (60.1)
Length of hospital stay, d												
1-2	281 (28.8)	203 (31.7)	78 (23.2)	101 (29.9)	54 (32.1)	47 (27.6)	180 (28.3)	149 (31.5)	31 (18.9)	2389 (25.7)	1793 (27.8)	596 (20.9)
3-6	470 (48.2)	321 (50.1)	149 (44.3)	146 (43.2)	78 (46.4)	68 (40.0)	324 (50.9)	243 (51.4)	81 (49.4)	3996 (43.0)	2660 (41.3)	1336 (46.9)
≥7	224 (23.0)	117 (18.3)	107 (31.8)	91 (26.9)	36 (21.4)	55 (32.4)	133 (20.9)	81 (17.1)	52 (31.7)	2912 (31.3)	1995 (30.9)	917 (32.2)
ICU admission^e^												
No	853 (87.5)	578 (90.2)	275 (81.8)	310 (91.7)	157 (93.5)	153 (90.0)	543 (85.2)	421 (89.0)	122 (74.4)	7415 (79.8)	5047 (78.3)	2368 (83.1)
Yes	122 (12.5)	63 (9.8)	59 (17.6)	28 (8.3)	11 (6.5)	17 (10.0)	94 (14.8)	52 (11.0)	42 (25.6)	1882 (20.2)	1401 (21.7)	481 (16.9)
Death^e^												
No	969 (99.4)	640 (99.8)	329 (97.9)	337 (99.7)	168 (100.0)	169 (99.4)	632 (99.2)	472 (99.8)	160 (97.6)	8999 (96.8)	6251 (96.9)	2748 (96.5)
Yes	6 (0.6)	1 (0.2)	5 (1.5)	1 (0.3)	0	1 (0.6)	5 (0.8)	1 (0.2)	4 (2.4)	298 (3.2)	197 (3.1)	101 (3.5)
Invasive mechanical ventilation^f^												
No	809 (83.0)	550 (85.8)	259 (77.1)	261 (77.2)	123 (73.2)	138 (81.2)	548 (86.0)	427 (90.3)	121 (73.8)	6503 (69.9)	4487 (69.6)	2016 (70.8)
Unknown	95 (9.7)	55 (8.6)	40 (11.9)	65 (19.2)	39 (23.2)	26 (15.3)	30 (4.7)	16 (3.4)	14 (8.5)	1526 (16.4)	961 (14.9)	565 (19.8)
Yes	71 (7.3)	36 (5.6)	35 (10.4)	12 (3.6)	6 (3.6)	6 (3.5)	59 (9.3)	30 (6.3)	29 (17.7)	1268 (13.6)	1000 (15.5)	268 (9.4)
ARDS^f^												
No	948 (97.2)	637 (99.4)	311 (92.6)	332 (98.2)	166 (98.8)	166 (97.6)	616 (96.7)	471 (99.6)	145 (88.4)	9030 (97.1)	6371 (98.8)	2659 (93.3)
Yes	27 (2.8)	4 (0.6)	23 (6.8)	6 (1.8)	2 (1.2)	4 (2.4)	21 (3.3)	2 (0.4)	19 (11.6)	267 (2.9)	77 (1.2)	190 (6.7)
Respiratory failure^f^												
No	732 (75.1)	564 (88.0)	168 (50.0)	223 (66.0)	149 (88.7)	74 (43.5)	509 (79.9)	415 (87.7)	94 (57.3)	5078 (54.6)	4032 (62.5)	1046 (36.7)
Yes	243 (24.9)	77 (12.0)	166 (49.4)	115 (34.0)	19 (11.3)	96 (56.5)	128 (20.1)	58 (12.3)	70 (42.7)	4219 (45.4)	2416 (37.5)	1803 (63.3)

Abbreviations: ARDS, acute respiratory distress syndrome; CLI, COVID-19–like illness.

^a^ Medical events with an encounter or discharge code consistent with CLI were included, using *International Classification of Diseases, Ninth Revision* and *International Statistical Classification of Diseases and Related Health Problems, Tenth Revision*. Four categories of codes were considered: (1) acute respiratory illness, including respiratory failure, viral or bacterial pneumonia, asthma exacerbation, influenza, and viral illness not otherwise specified; (2) nonrespiratory CLI diagnoses including cause-unspecified gastroenteritis, thrombosis, and acute myocarditis; (3) respiratory signs and symptoms consistent with CLI illness, including hemoptysis, cough, dyspnea, painful respiration, or hypoxemia; and (4) signs and symptoms of acute febrile illness. One code in any of the 4 categories was sufficient for inclusion. Clinician-ordered molecular assays (eg, real-time reverse transcription–polymerase chain reaction) for SARS-CoV-2 occurring 14 or fewer days before to less than 72 hours after the encounter date were included.

^b^ Partners contributing data on medical events were in California (estimated start date of Delta predominance, June 23; estimated start date of Omicron predominance, December 21), Colorado (estimated start date of Delta predominance, June 3; estimated start date of Omicron predominance, December 19), Indiana (estimated start date of Delta predominance, July 3; estimated start date of Omicron predominance, December 26), Minnesota and Wisconsin (estimated start date of Delta predominance, July 1; estimated start date of Omicron predominance, December 25), New York (estimated start date of Delta predominance, June 30; estimated start date of Omicron predominance, December 18), Oregon (estimated start date of Delta predominance, June 30; estimated start date of Omicron predominance, December 24), Texas (estimated start date of Delta predominance, September 11; estimated start date of Omicron predominance, December 16), Utah (estimated start date of Delta predominance, June 1; estimated start date of Omicron predominance, December 24), and Washington (estimated start date of Delta predominance, June 30; estimated start date of Omicron predominance, December 24). The study period began in September 2021 for partners located in Texas.

^c^ Hospitalization encounters where there was no indication of delivery at hospital discharge.

^d^ Hospitalization encounters where there was documented evidence of delivery at hospital discharge.

^e^ Event occurred at any point during the hospital admission.

^f^ Condition documented with *International Classification of Diseases *codes at hospital discharge.

**Figure 2.**
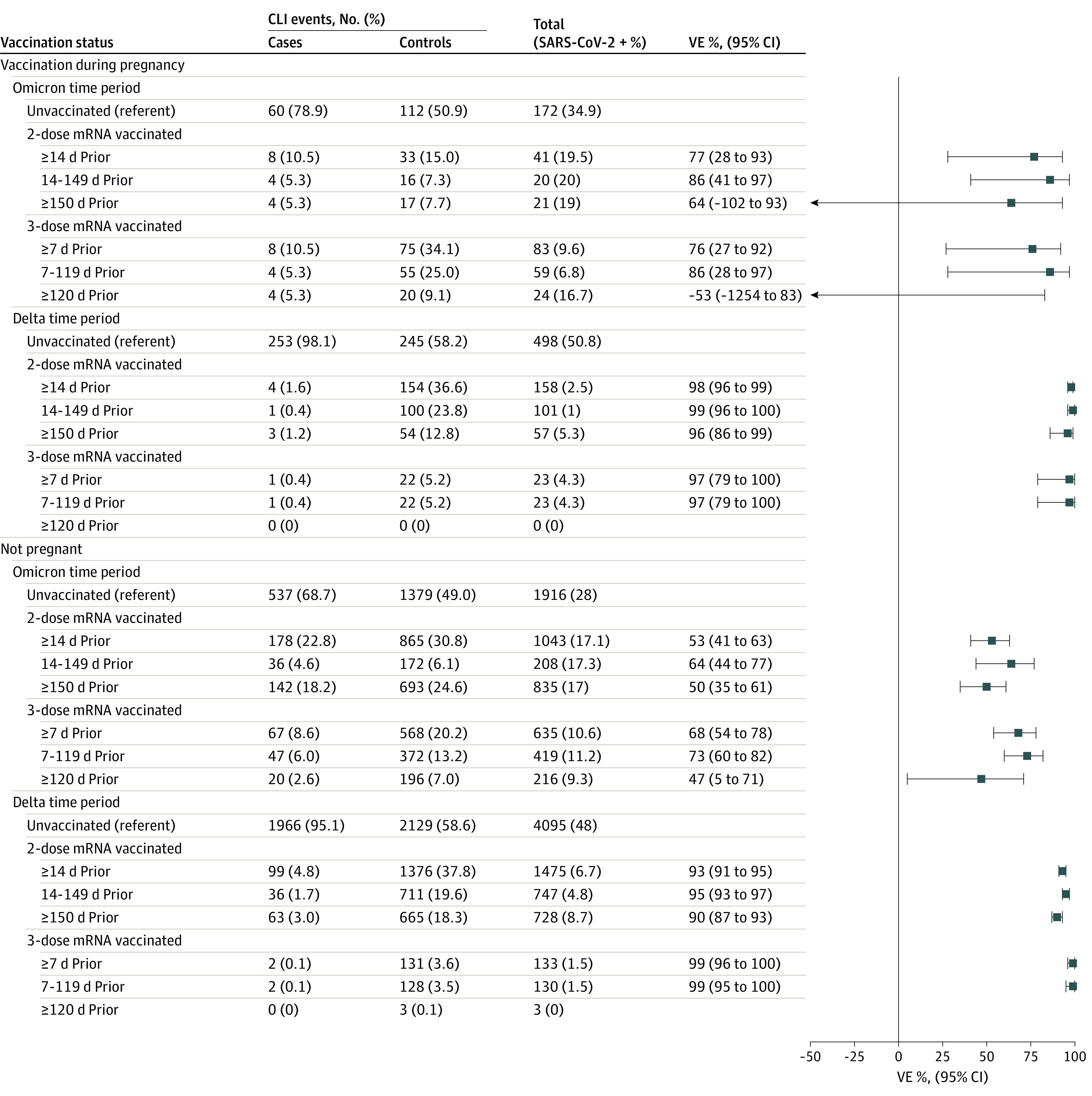
mRNA COVID-19 Vaccine Effectiveness (VE) Against Laboratory-Confirmed COVID-19–Associated Hospitalizations Among Pregnant People and Nonpregnant Women, VISION Network, 10 States, June 1, 2021, to June 2, 2022 The VISION Network includes Baylor Scott & White Health (Texas), Columbia University Irving Medical Center (New York), HealthPartners (Minnesota and Wisconsin), Intermountain Healthcare (Utah), Kaiser Permanente Northern California (California), Kaiser Permanente Northwest (Oregon and Washington), Regenstrief Institute (Indiana), and University of Colorado (Colorado). Vaccinated pregnant people included in this analysis received at least their most recent dose during pregnancy; among individuals with 3 doses, only 2 received the third dose less than 150 days after the second dose. Among vaccinated nonpregnant women with 3 doses, 130 in the Delta period and 43 in the Omicron period received the third dose less than 150 days after the second dose. VE was calculated as described in the Methods section. VE could not be calculated for the individuals during the Delta period who received 3 doses of mRNA vaccine 120 or more days prior to hospitalization because the stratum included fewer than 20 encounters with prior vaccination. CLI indicates COVID-19–like illness.

During Delta variant predominance, the estimated VE of 2 mRNA vaccine doses 14 to 149 days prior against COVID-19–associated hospitalization was 99% (95% CI, 96%-100%) (Figure 2); for 2 doses 150 or more days prior, it was 96% (95% CI, 86%-99%). The estimated VE of 3 doses 7 to 119 days prior was 97% (95% CI, 79%-100%); the sample was not sufficient to estimate VE for 3 doses 120 or more days or more prior. During Omicron variant predominance, the estimated VE of 2 doses 14 to 149 days prior was 86% (95% CI, 41% to 97%); for 2 doses 150 or more days prior it was 64% (95% CI, −102% to 93%). The estimated VE of 3 doses 7 to 119 and 120 or more days prior was 86% (95% CI, 28% to 97%) and −53% (95% CI, −1254% to 83%), respectively.

A sensitivity analysis among pregnant persons regardless of timing of vaccination (ie, not excluding those who received all doses before pregnancy) yielded VE estimates that were not significantly different from the primary results (eTables 4 and 5 and eFigures 2 and 3 in the Supplement). VE estimates among nonpregnant women were also not significantly different from estimates among pregnant persons, although point estimates varied and confidence intervals were narrower in nonpregnant women because of larger sample size (eTables 6 and 7 in the Supplement and Figures 1 and 2). VE estimates limited to pregnant people with no prior documented SARS-CoV-2 infection were not significantly different from estimates that included all eligible regardless of prior infection (eTables 8 and 9 and eFigures 4 and 5 in the Supplement).

## Discussion

This study found high maternal protection against medically attended COVID-19 in the Delta period for 2 and 3 mRNA doses and highlighted the importance of a booster dose for both ED/UC and hospitalization end points during Omicron predominance. As with other evaluations of mRNA COVID-19 vaccines in adults,^16^ estimated effectiveness against ED/UC encounters was generally lower than for hospitalizations, and this difference was most marked for 2 doses during the Omicron period. Additionally, decreased protection of the primary series after 150 or more days since the second dose against the hospitalization end point was more evident during the Omicron period than during the earlier Delta period, highlighting the importance of booster doses among pregnant persons, in line with current recommendations.^4,5^

Prior assessments of VE among pregnant women preceded booster recommendations for this population, focused primarily on the pre-Delta period, had limited sample size to assess more serious COVID-19 end points, and evaluated limited durations since the second dose.^7,8,9,10,11^ These prior studies suggested the mRNA vaccine primary series among pregnant people was associated with strong protection against COVID-19 disease and SARS-CoV-2 infections in the period shortly after the second dose, similar to VE assessments among general adult populations. The estimates of effectiveness among pregnant women presented in this study provide more current and complete evidence, and continue to suggest that mRNA vaccine administration during pregnancy does not alter vaccine performance despite immune differences between pregnant and nonpregnant people.^17^ The patterns we observed among VE estimates for pregnant people were similar to those for women aged 18 to 45 years who were not pregnant at the time of their CLI episode, although the estimates for this latter group, due to larger sample size, had tighter confidence bounds; they were also consistent with VE estimates among general adult populations from other studies.^16,18,19^ The estimated VE among pregnant people receiving at least 1 dose during pregnancy was also similar to that among pregnant people vaccinated before or during pregnancy.

The findings reported here that COVID-19 maternal vaccination appears to protect pregnant people against medically attended COVID-19 is important given accruing evidence of increased risks of severe maternal morbidity and mortality associated with COVID-19 during pregnancy.^1,2,3,20^ Although two-thirds of CLI hospitalizations were associated with delivery, COVID-19 hospitalizations among delivering pregnant people were similar in terms of length of stay (>70% greater than 2 days) and had higher proportions of mechanical ventilation and intensive care unit admission than nondelivery and nonpregnant COVID-19 hospitalizations, suggesting that the outcome used for this study did not reflect incidental infection among women admitted for delivery. Moreover, among COVID-19–associated hospitalizations during which delivery occurred, 54.9% delivered preterm, highlighting the frequency of adverse pregnancy outcomes among pregnant women with COVID-19. While we were able to demonstrate apparent significant and substantial protection against COVID-19–associated hospitalizations and ED/UC visits, we did not have a sufficient sample size to assess whether maternal vaccination was associated with attenuation of the COVID-19 course among hospitalized pregnant people with COVID-19, as has been described for mRNA vaccines in the general adult population.^21^ Additional evaluations are also needed for a full assessment of waning protection after a booster dose, which has been observed among nonpregnant adults.^16^

Direct maternal benefits of COVID-19 vaccines add to the accumulating picture of the broader benefits that extend beyond the pregnant person to the fetus and newborn. A recent test-negative design study among infants younger than 6 months hospitalized with CLI in the United States^22^ found that maternal receipt of 2 doses of mRNA vaccine during pregnancy was less common among polymerase chain reaction– or antigen-positive case infants (16%) than among polymerase chain reaction– or antigen-negative control infants (32%), yielding an estimated VE for young infants of 61% (95% CI, 31%-78%). Additionally, a recent safety evaluation of a cohort of more than 46 000 live births in the United States^23^ found 2 doses of mRNA vaccine in the third trimester to be associated with reduced risk of preterm delivery (adjusted hazard ratio. 0.82; 95% CI, 0.72-0.94), and a large safety study in Ontario, Canada, that focused on a range of pregnancy outcomes and newborn health indicators^24^ suggested that maternal vaccination might protect against low Apgar scores (an assessment of infant condition soon after birth) and neonatal intensive care unit admission. Pregnant people in the United States have lagged behind similarly aged nonpregnant adults in primary series and booster dose coverage.^6^ The growing evidence of the benefits of vaccination during pregnancy, along with postlicensure safety information, addresses important knowledge gaps that arose from the exclusion of pregnant individuals from mRNA vaccine clinical trials and could improve acceptance of COVID-19 vaccination among pregnant people.^25,26^

### Limitations

This analysis is subject to several limitations. First, the methods used to identify pregnancy and determine gestational age were not standardized across sites; misclassification of pregnancy status at the time of vaccination and CLI medical encounter may have occurred. Second, the threshold for ED/UC care-seeking during pregnancy may be lower than for nonpregnant adults and thus the ED/UC outcome may capture milder infection events. Third, clinicians may be more inclined to admit unvaccinated than vaccinated patients (regardless of pregnancy) because of the potential for developing severe disease, which could theoretically bias toward a higher VE for the hospitalization outcome. Fourth, the analysis did not exclude immunocompromised individuals, for whom an additional primary dose of mRNA vaccine is recommended 4 weeks after dose 2; however, immunocompromised status was uncommon among pregnant people with CLI (<1% of ED/UC visits and <5% of hospitalizations). Fifth, COVID-19 vaccine coverage among pregnant people, particularly for a third dose, was low; the potential for bias arising from differences between vaccinated and unvaccinated individuals that affects all observational VE studies may be even more relevant for this population. Although weights for propensity-to-be-vaccinated and relevant covariates were included in VE estimation models, there is still potential for residual or unmeasured confounding. Sixth, case counts during Omicron predominance were lower than during Delta predominance, resulting in more limited power for VE estimation during Omicron, particularly for the hospitalization outcome. Seventh, due to limited sample size we could not adjust VE estimates for county clusters within health systems but did adjust for the catchment region of each health system.^12^ Eighth, the characteristics of pregnant people included in VISION may not be generalizable to the pregnant population in the United States, although the VISION network covers 8 health systems across 10 states.

## Conclusions

In this study, maternal mRNA COVID-19 vaccination, including booster dose, was associated with protection against medically attended COVID-19. VE estimates were higher against COVID-19–associated hospitalization than ED/UC visits and lower against Omicron than Delta, and protection waned over time, particularly during Omicron predominance.

## Notes

Supplement. **eTable 1.** VISION Network Site Methods for Ascertainment of Gestational Age at Vaccination and the CLI Event and Whether the CLI Event Was Associated With Delivery **eTable 2.** Acute COVID-19–Like Illness Categories and Related *International Classification of Diseases*, *9th* and *10th Revision* (*ICD*) Discharge Codes for Hospitalization and ED/UC Visits **eTable 3. **VISION Partner Site-Specific Start Dates for Delta and Omicron Predominance and Geographic Region **eTable 4.** Characteristics of Hospitalizations Among Pregnant People With COVID-19–Like Illness by COVID-19 mRNA Vaccination Status (Regardless of Pregnancy Status at Time of Vaccination) and SARS-CoV-2 Test Result, 10 States, June 1, 2021, to June 2, 2022 **eTable 5.** Characteristics of Emergency Department and Urgent Care Encounters Among Pregnant People With COVID-19–Like Illness by COVID-19 mRNA Vaccination Status (Regardless of Pregnancy Status at Time of Vaccination) and SARS-CoV-2 Test Result, 10 States, June 1, 2021, to June 2, 2022 **eTable 6. **Characteristics of COVID-19–Like Illness Hospitalizations Among Nonpregnant Women Aged 18 to 45 Years With COVID-19–Like Illness by COVID-19 BNT162b2 Vaccination Status and SARS-CoV-2 Test Result, 10 States, June 1, 2021, to June 2, 2022 **eTable 7. **Characteristics of Emergency Department and Urgent Care Encounters Among Nonpregnant Women Aged 18 to 45 Years With COVID-19–Like Illness by COVID-19 BNT162b2 Vaccination Status and SARS-CoV-2 Test Result, 10 States, June 1, 2021, to June 2, 2022 **eTable 8.** Characteristics of Hospitalizations Among Pregnant People With COVID-19–Like Illness Who Did Not Have a Prior Positive SARS-CoV-2 Infection by COVID-19 mRNA Vaccination Status, VISION Network, 10 States, June 1, 2021–June 2, 2022 **eTable 9.** Characteristics of Emergency Department and Urgent Care Encounters Among Pregnant People With COVID-19–Like Illness Who Did Not Have a Prior Positive SARS-CoV-2 Infection by COVID-19 mRNA Vaccination Status, VISION Network, 10 States, June 1, 2021 to June 2, 2022 **eFigure 1.** Flow Figure for Hospitalizations and ED/UC Visits Among Pregnant People (Unvaccinated or With 1 Vaccine Dose Received During Pregnancy) **eFigure 2.** mRNA COVID-19 Vaccine Effectiveness Against Laboratory-Confirmed COVID-19–Associated Emergency Department and Urgent Care Encounter Among Pregnant People Regardless of Pregnancy Status at Time of Vaccination, VISION Network, 10 States, June 1, 2021, to June 2, 2022 **eFigure 3.** mRNA COVID-19 Vaccine Effectiveness Against Laboratory-Confirmed COVID-19–Associated Hospitalizations Among Pregnant People Regardless of Pregnancy Status at Time of Vaccination, VISION Network, 10 States, June 1, 2021, to June 2, 2022 **eFigure 4.** mRNA COVID-19 Vaccine Effectiveness Against Laboratory-Confirmed COVID-19–Associated Emergency Department and Urgent Care Encounter Among Pregnant People Who Did Not Have a Prior Positive SARS-CoV-2 Infection, VISION Network, 10 States, June 1, 2021, to June 2, 2022 **eFigure 5.** mRNA COVID-19 Vaccine Effectiveness Against Laboratory-Confirmed COVID-19–Associated Hospitalizations Among Pregnant People Who Did Not Have a Prior Positive SARS-CoV-2 Infection, VISION Network, 10 States, June 1, 2021, to June 2, 2022
