# Supplementary material for: Estimation of COVID-19 mRNA Vaccine Effectiveness Against Medically Attended COVID-19 in Pregnancy During Periods of Delta and Omicron Variant Predominance in the United States
Source: JAMA Netw Open. 2022 Sep 26;5(9):e2233273. doi: 10.1001/jamanetworkopen.2022.33273 (PMC9513651; doi:10.1001/jamanetworkopen.2022.33273)
Supplement: Supplement. — eTable 1. VISION Network Site Methods for Ascertainment of Gestational Age at Vaccination and the CLI Event and Whether the CLI Event Was Associated With Delivery eTable 2. Acute COVID-19–Like Illness Categories and Related International Classification of Diseases, 9th and 10th Revision (ICD) Discharge Codes for Hospitalization and ED/UC Visits eTable 3. VISION Partner Site-Specific Start Dates for Delta and Omicron Predominance and Geographic Region eTable 4. Characteristics of Hospitalizations Among Pregnant People With COVID-19–Like Illness by COVID-19 mRNA Vaccination Status (Regardless of Pregnancy Status at Time of Vaccination) and SARS-CoV-2 Test Result, 10 States, June 1, 2021, to June 2, 2022 eTable 5. Characteristics of Emergency Department and Urgent Care Encounters Among Pregnant People With COVID-19–Like Illness by COVID-19 mRNA Vaccination Status (Regardless of Pregnancy Status at Time of Vaccination) and SARS-CoV-2 Test Result, 10 States, June 1, 2021, to June 2, 2022 eTable 6. Characteristics of COVID-19–Like Illness Hospitalizations Among Nonpregnant Women Aged 18 to 45 Years With COVID-19–Like Illness by COVID-19 BNT162b2 Vaccination Status and SARS-CoV-2 Test Result, 10 States, June 1, 2021, to June 2, 2022 eTable 7. Characteristics of Emergency Department and Urgent Care Encounters Among Nonpregnant Women Aged 18 to 45 Years With COVID-19–Like Illness by COVID-19 BNT162b2 Vaccination Status and SARS-CoV-2 Test Result, 10 States, June 1, 2021, to June 2, 2022 eTable 8. Characteristics of Hospitalizations Among Pregnant People With COVID-19–Like Illness Who Did Not Have a Prior Positive SARS-CoV-2 Infection by COVID-19 mRNA Vaccination Status, VISION Network, 10 States, June 1, 2021–June 2, 2022 eTable 9. Characteristics of Emergency Department and Urgent Care Encounters Among Pregnant People With COVID-19–Like Illness Who Did Not Have a Prior Positive SARS-CoV-2 Infection by COVID-19 mRNA Vaccination Status, VISION Network, 10 States, June 1, 2021 [file jamanetwopen-e2233273-s001.pdf]

Supplemental Online Content

Schrag SJ, Verani JR, Dixon BE, et al. COVID-19 mRNA vaccination effectiveness against medically attended COVID-19 in pregnancy during periods of Delta and Omicron variant predominance in the United States. *JAMA Netw Open.* 2022;5(9):e2233273. doi:10.1001/jamanetworkopen.2022.33273

eTable 1. VISION Network Site Methods for Ascertainment of Gestational Age at Vaccination and the CLI Event and Whether the CLI Event Was Associated With Delivery

eTable 2. Acute COVID-19–Like Illness Categories and Related *International Classification of Diseases*, *9th* and *10th Revision* (*ICD*) Discharge Codes for Hospitalization and ED/UC Visits

eTable 3. VISION Partner Site-Specific Start Dates for Delta and Omicron Predominance and Geographic Region

eTable 4. Characteristics of Hospitalizations Among Pregnant People With COVID-19–Like Illness by COVID-19 mRNA Vaccination Status (Regardless of Pregnancy Status at Time of Vaccination) and SARS-CoV-2 Test Result, 10 States, June 1, 2021, to June 2, 2022

eTable 5. Characteristics of Emergency Department and Urgent Care Encounters Among Pregnant People With COVID-19–Like Illness by COVID-19 mRNA Vaccination Status (Regardless of Pregnancy Status at Time of Vaccination) and SARS-CoV-2 Test Result, 10 States, June 1, 2021, to June 2, 2022

eTable 6. Characteristics of COVID-19–Like Illness Hospitalizations Among Nonpregnant Women Aged 18 to 45 Years With COVID-19–Like Illness by COVID-19 BNT162b2 Vaccination Status and SARS-CoV-2 Test Result, 10 States, June 1, 2021, to June 2, 2022

eTable 7. Characteristics of Emergency Department and Urgent Care Encounters Among Nonpregnant Women Aged 18 to 45 Years With COVID-19–Like Illness by COVID-19 BNT162b2 Vaccination Status and SARS-CoV-2 Test Result, 10 States, June 1, 2021, to June 2, 2022

eTable 8. Characteristics of Hospitalizations Among Pregnant People With COVID-19–Like Illness Who Did Not Have a Prior Positive SARS-CoV-2 Infection by COVID-19 mRNA Vaccination Status, VISION Network, 10 states, June 1, 2021–June 2, 2022

eTable 9. Characteristics of Emergency Department and Urgent Care Encounters Among Pregnant People With COVID-19–Like Illness Who Did Not Have a Prior Positive SARS-CoV-2 Infection by COVID-19 mRNA Vaccination Status, VISION Network, 10 States, June 1, 2021 to June 2, 2022

eFigure1. Flow Figure for Hospitalizations and ED/UC Visits Among Pregnant People (Unvaccinated or With 1 Vaccine Dose Received During Pregnancy)

eFigure 2. mRNA COVID-19 Vaccine Effectiveness Against Laboratory-Confirmed COVID-19–Associated Emergency Department and Urgent Care Encounter Among Pregnant People

© 2022 Schrag SJ et al. *JAMA Network Open.*

Regardless of Pregnancy Status at Time of Vaccination, VISION Network, 10 States, June 1, 2021, to June 2, 2022

eFigure 3. mRNA COVID-19 Vaccine Effectiveness Against Laboratory-Confirmed COVID-19–Associated Hospitalizations Among Pregnant People Regardless of Pregnancy Status at Time of Vaccination, VISION Network, 10 states, June 1, 2021, to June 2, 2022

eFigure 4. mRNA COVID-19 Vaccine Effectiveness Against Laboratory-Confirmed COVID-19–Associated Emergency Department and Urgent Care Encounter Among Pregnant People Who Did Not Have a Prior Positive SARS-CoV-2 Infection, VISION Network, 10 States, June 1, 2021, to June 2, 2022

eFigure 5. mRNA COVID-19 Vaccine Effectiveness Against Laboratory-Confirmed COVID-19–Associated Hospitalizations Among Pregnant People Who Did Not Have a Prior Positive SARS-CoV-2 Infection, VISION Network, 10 States, June 1, 2021, to June 2, 2022

This supplemental material has been provided by the authors to give readers additional information about their work.

© 2022 Schrag SJ et al. *JAMA Network Open.*

eTable 1. VISION Network Site Methods for Ascertainment of Gestational Age at Vaccination and the CLI Event and Whether the CLI Event Was Associated With Delivery

Site	Source of information to estimate gestational age at vaccination and CLI event	Source of information used to ascertain whether the CLI event was associated with delivery
KPNW	Kaiser Permanente Center for Effectiveness and Safety Research (CESR) pregnancy algorithm, which is an algorithm related to the Vaccine Safety Datalink (VSD) algorithm[1,2] that also incorporates additional laboratory and ultrasound information.	Kaiser Permanente Center for Effectiveness and Safety Research (CESR) pregnancy algorithm, which is an algorithm related to the Vaccine Safety Datalink (VSD) algorithm[1,2] that also incorporates additional laboratory and ultrasound information.
HP	VSD algorithm[1,2]	VSD algorithm[1,2]
KPNC	VSD algorithm[1,2]	VSD algorithm[1,2]
IM	Health system creates pregnancy data tables for active members that include estimated delivery date. Estimated delivery date is used to estimate a pregnancy start date which is then used to estimate gestational age associated with any subsequent events during pregnancy. The estimated delivery date is populated with an actual delivery date when delivery occurs within the hospital system.	Health system captures a delivery date for people who deliver within the hospital system.
UCO	A pregnancy flag and pregnancy start and end dates are captured as fields in the EPIC chart; these fields are pulled into pregnancy data tables and used to estimate gestational age	A delivery flag is included in the EPIC chart.
CU	The health system captures data field for pregnancy end dates. The estimated date of delivery date is used to calculate pregnancy begin date and gestational age associated with any subsequent events during pregnancy.	The health system has a flag for delivery.
RG	RG uses a Health Information Exchange (HIE) network for their data source rather than an Electronic Health Records (EHR) system[3]. Pregnancy flag created by screening all health system encounters +/- 30 days of the CLI event for ICD codes related to pregnancy or delivery (ICD code list based on VSD algorithm[1,2]) GA is determined using pregnancy start date, if captured. If not, any gestational age-related ICD codes captured +/- 7 days of the CLI event were used to estimate a pregnancy begin date which were used to estimate gestational age at the time of vaccination and CLI event. Gestational age was available for ~55% of records.	If the HIE network captured a delivery date for the patient, this date is used. If a delivery date is not available, the ICD codes associated with the CLI event were screened for delivery indicators.
BSWH	The health system creates pregnancy data tables for active patients that include Estimated Delivery Date (EDD). EDD is used to estimate a Pregnancy Start Date which is used to estimate Gestational Age associated with any subsequent encounters during pregnancy. If the EDD is missing, the last menstrual period (LMP) or the antenatal visit ICD codes that include gestational age are used to estimate the EDD.	The health system captures a Delivery Date for deliveries within the hospital system.

1. Naleway AL, Gold R, Kurosky S, et al. Identifying pregnancy episodes, outcomes, and mother-infant pairs in the Vaccine Safety Datalink. Vaccine 2013;31(27):2898-903. (In eng). DOI: 10.1016/j.vaccine.2013.03.069.
2. Naleway AL, Crane B, Irving SA, et al. Vaccine Safety Datalink infrastructure enhancements for evaluating the safety of maternal vaccination. Ther Adv Drug Saf 2021;12:20420986211021233. (In eng). DOI: 10.1177/20420986211021233.
3. Overhage JM. The Indiana Health Information Exchange. In: Dixon BE, editor. Health Information Exchange: Navigating and Managing a Network of Health Information Systems. 1 ed. Waltham, MA: Academic Press; 2016. p. 267-79.

© 2022 Schrag SJ et al. *JAMA Network Open.*

eTable 2. Acute COVID-19–Like Illness Categories and Related International Classification of Diseases, 9th and 10th Revision (ICD) Discharge Codes for Hospitalization and ED/UC Visits

Disease Condition	ICD-10 Codes	ICD-9 Codes
COVID-19 Pneumonia		
Pneumonia due to SARS-associated coronavirus	J12.81	N/A
Pneumonia due to coronavirus disease 2019	J12.82	N/A
Influenza Pneumonia		
Influenza due to identified novel influenza A virus with pneumonia	J09.X1	488.81
Influenza due to other identified influenza virus with pneumonia	J10.0	N/A
Influenza due to other identified influenza virus with unspecified type of pneumonia	J10.00	487.0
Influenza due to other identified influenza virus with the same other identified influenza virus pneumonia	J10.01	487.0
Influenza due to other identified influenza virus with other specified pneumonia	J10.08	487.0, 488.11
Influenza due to unidentified influenza virus with pneumonia	J11.0	N/A
Influenza due to unidentified influenza virus with unspecified type of pneumonia	J11.00	487.0
Influenza due to unidentified influenza virus with specified pneumonia	J11.08	487.0
Influenza with pneumonia	N/A	487*
Other Viral Pneumonia	J12*	480*
Bacterial and Other Pneumonia		
Streptococcus pneumoniae pneumonia	J13	481
Hemophilus influenzae pneumonia	J14	482.2
Other bacterial pneumonia	J15*	482*
Pneumonia due to other specified organism	J16*	483*
Pneumonia in infectious diseases classified elsewhere	J17	484*
Pneumonia, unspecified organism	J18*	486
Influenza Disease	J09*, J10.1, J10.2, J10.8*, J11.1, J11.2, J11.8*	488*
Acute respiratory distress syndrome	J80	518.82
COPD with acute exacerbation	J44.1	491.21
Asthma acute exacerbation	J45.21, J45.22, J45.31, J45.32, J45.41, J45.42, J45.51, J45.52, J45.901, J45.902	493.01, 493.02, 493.11, 493.12, 493.21, 493.22, 493.91, 493.92
Respiratory failure		
Acute respiratory failure	J96.0*	518.81
Acute and chronic respiratory failure	J96.2*	518.84
Respiratory arrest	R09.2	799.1

© 2022 Schrag SJ et al. *JAMA Network Open.*

Other acute lower respiratory tract infections		
Acute bronchitis	J20*	466.0
Acute bronchiolitis	J21*	466.1*
Unspecified acute lower respiratory infection	J22	519.8
Bronchitis, not specified as acute or chronic	J40	490
COPD with acute lower respiratory infection	J44.0	491.22
Simple and mucopurulent chronic bronchitis	J41*	491*
Unspecified chronic bronchitis	J42	491.9
Emphysema	J43*	492*
Bronchiectasis	J47*	494*
Abscess of lung and mediastinum	J85	513
Gangrene and necrosis of lung	J85.0	N/A
Abscess of lung without pneumonia	J85.2	513.0
Abscess of mediastinum	J85.3	513.1
Abscess of lung with pneumonia	J85.1	513.0
Pyothorax	J86*	510*
Acute and chronic sinusitis	J01*, J32*	461*,473*
Acute upper respiratory tract infections	J00*, J02*-J06*	460*, 462*-465*
Signs and symptoms of acute respiratory illness		
Hemoptysis	R04.2	786.3
Cough	R05, R05.1, R05.2, RO5.4, RO5,8, RO5.9	786.2
Dyspnea unspecified	R06.00	786.09
Shortness of breath	R06.02	786.05
Acute respiratory distress	R06.03	N/A
Stridor	R06.1	786.1
Wheezing	R06.2	786.07
Other abnormalities of breathing	R06.8	N/A
Apnea, not elsewhere classified	R06.81	786.03
Tachypnea, NEC	R06.82	786.06
Other abnormalities of breathing/ Other symptoms involving head & neck	R06.89	784.99
Other dyspnea and respiratory abnormality	N/A	786.09
Other symptoms involving respiratory system and chest	N/A	786.9
Chest pain on breathing/ painful respiration	R07.1	786.52
Asphyxia and hypoxemia	R09.0*	N/A
Asphyxia	R09.01	799.01
Hypoxemia	R09.02	799.02
Pleurisy	R09.1	511.0
Respiratory arrest	R09.2	799.1
Abnormal sputum	R09.3	786.4

© 2022 Schrag SJ et al. *JAMA Network Open.*

Other specified symptoms and signs involving the circulatory and respiratory systems	R09.8*	478.19, 784.91, 786.7
Signs and symptoms of acute febrile illness		
Fever	R50*	N/A
Fever presenting with conditions classified elsewhere	R50.81	780.61
Fever unspecified	R50.9	780.60
Chills (without fever)	R68.83	780.64
Signs and symptoms of acute non-respiratory illness**		
Diarrhea	R19.7	787.91
Disturbance of smell and taste	R43*	N/A
Unspecified disturbances of smell and taste	R43.9	781.1, V41.5
Headache	R51*	784.0
Myalgia	M79.1*	729.1
Sepsis - Symptoms and signs specifically associated with systemic inflammation and infection	R65*	785.52
Other malaise	R53.81	780.79
Other fatigue	R53.83	780.79
Shock, unspecified	R57.9	785.5
Debility unspecified	N/A	799.3
Altered level of consciousness / altered mental status	R41.82, R40*	780.97, 780.0*
Weakness	R53.1	780.79
Nausea and Vomiting	R11.*	787*
Rash and other nonspecific skin eruption	R21.*	782.1
Abdominal pain	R10*	789*

* Includes all sub-codes
** Records for which these codes were the sole CLI criteria visit were excluded from the analysis of CLI hospitalizations (but included for the analysis of CLI ED/UC encounter)

© 2022 Schrag SJ et al. *JAMA Network Open.*

eTable 3. VISION Partner Site-Specific Start Dates for Delta and Omicron Predominance and Geographic Region

VISION Partner	Delta Predominance Start Date	Omicron Predominance Start Date	Composition of geographic areas defined for each VISION partner site
Baylor Scott & White Health (BSWH)	September 11, 2021 (study period begin date)	December 16, 2021	State: Texas Region 1: Anderson, Andrews, Angelina, Archer, Armstrong, Bailey, Baylor, Borden, Bowie, Briscoe, Brown, Callahan, Camp, Carson, Cass, Castro, Cherokee, Childress, Clay, Cochran, Coke, Coleman, Collin, Collingsworth, Comanche, Cooke, Cottle, Crosby, Dallam, Dallas, Dawson, Deaf Smith, Delta, Denton, Dickens, Donley, Eastland, Ector, Ellis, Erath, Fannin, Fisher, Floyd, Foard, Franklin, Freestone, Gaines, Glasscock, Gray, Grayson, Gregg, Grimes, Hale, Hall, Hansford, Hardeman, Harrison, Hartley, Haskell ,Hemphill, Henderson, Hockley, Hood, Hopkins, Houston, Howard, Hunt, Hutchinson, Jack, Jasper, Johnson, Hones, Kaufman, Kent, King, Knox, Lamar, Lamb, Leon, Lipscomb, Lubbock, Lynn, Madison, Marion, Martin, Midland, Mitchell, Montague, Montgomery, Moore, Morris, Motley, Nacogdoches, Navarro, Newton, Nolan, Ochiltree, Oldman, Palo Pinto, Panola, Parker, Parmer, Polk, Potter, Raines, Randall, Red River, Roberts, Rockwall, Runnels, Rusk, Sabine, San Augustine, San Jacinto, Scurry, Shackleford, Shelby, Sherman, Smith, Somervell, Stephens, Sterling, Stonewall, Swisher, Tarrant, Taylor, Terry, Throckmorton, Titus, Trinity, Tyler, Upshur, Van Zandt, Walker, Waller, Wheeler, Wichita, Wilbarger, Winkler, Wise, Wood, Yoakum and Young counties Region 2: Aransas, Atascosa, Austin, Bandera, Bastrop, Bee, Bell, Bexar, Blanco, Bosque, Brazoria, Brazos, Brewster, Brooks, Burleson, Burnet, Caldwell, Calhoun, Cameron, Chambers, Colorado, Comal, Concho, Coryell, Crace, Crockett, Culberson, Dewitt, Dimmit, Duval, Edwards, El Paso, Falls, Fayette, Fort Bend, Frio, Galveston, Gillespie, Goliad, Gonzales, Guadalupe, Hamilton, Hardin, Harris, Hays, Hildago, Hill, Hudspeth, Irion, Jackson, Jeff Davis, Jefferson, Jim Hogg, Jim Wells, Karnes, Kendall, Kenedy, Kerr, Kimble, Kinney, Kelberg, La Salle, Lampasas, Lavaca, Lee, Liberty, Limestone, Live Oak, Llano, Loving, Mason, Matagorda, Maverick, McCulloch, McClennan, McMullen, Medina, Menard, Milam, Mills, Nueces, Orange, Pecos, Presidio, Reagan, Real, Reeves, Refugio, Robertson, San Patricio, San Saba, Schleicher, Starr, Sutton, Terrell, Tom Green, Travis, Upton, Uvalde, Val Verde, Victoria, Ward, Washington, Webb, Wharton, Willacy, Williamson, Wilson, Zapata, and Zavala counties
Columbia University (CUIMC)	June 30, 2021	December 18, 2022	State: New York Region 1: Bronx county, Kings county, New York County, Queens county, Richmond County
HealthPartners (HP)	July 1, 2021	December 25, 2021	State: Minnesota Region 1: Hennepin and Ramsey counties; Region 2: Polk, St. Croix and Washington, Mcleod and Renville counties
Intermountain Healthcare (IH)	June 1, 2021	December 24, 2021	State: Utah Region 1: Box Elder county and Cache counties; Region 2: Davis County; Region 3: Tooele and Salt Lake counties; Region 4: Utah county; Region 5: Summit and Wasatch counties;

© 2022 Schrag SJ et al. *JAMA Network Open.*

			Region 6: Weber County; Region 7: Sanpete, Sevier and Millard counties; Region 8: Garfield, Iron and Washington counties.
Kaiser Permanente Northern California (KPNC)	June 23, 2021	December 21, 2021	State: California Region 1: Fresno County; Region 2: Stanislaus county; Region 3: Placer and Sacramento counties; Region 4: Alameda, Contra Costa, Marin, San Francisco, and San Mateo counties; Region 5: Santa Clara county; Region 6: Sonoma county; Region 7: San Joaquin county; Region 8: Solano county
Kaiser Permanente Northwest (KPNW)	June 30, 2021	December 24, 2021	State: Oregon Region 1: Multnomah, Washington, Clackamas, Clatsop, and Yamhill counties; State: Washington Region 2: King, Chelan, Clark, Pacific, Spokane and Cowlitz counties; Oregon Region 3: Benton, Deschutes, Harney, Lane, Linn, Marion, Polk, Umatilla, and Wasco counties
Regenstrief Institute (RG)	July 3, 2021	December 26, 2021	State: Indiana Region 1: Jasper, Lake, La Porte, Newton, Porter; Region 2: Elkhart, Fulton, Kosciusko, Marshall, Pulaski, St. Joseph, Starke; Region 3: Adams, Allen, DeKalb, Huntington, Lagrange, Miami, Noble, Steuben, Wabash, Wells, Whitley; Region 4: Benton, Carroll, Cass, Clinton, Fountain, Tippecanoe, Montgomery, Warren, White; Region 5: Boone, Johnson, Hamilton, Hancock, Hendricks, Marion, Morgan, Shelby; Region 6: Blackford, Delaware, Fayette, Grant, Henry, Howard, Jay, Madison, Randolph, Rush, Tipton, Union, Wayne; Region 7: Clay, Greene, Owen, Parke, Putnam, Sullivan, Vermillion, Vigo; Region 8: Bartholomew, Brown, Jackson, Lawrence, Monroe, Orange, Washington; Region 9: Clark, Dearborn, Decatur, Floyd, Franklin, Harrison, Jefferson, Jennings, Ohio, Ripley, Scott, Switzerland; Region 10: Crawford, Daviess, Dubois, Gibson, Knox, Martin, Spencer, Perry, Pike, Posey, Vanderburgh, Warrick; Region 11: all encounters missing county information
University of Colorado (UCO)	June 3, 2021	December 19, 2021	State: Colorado Region 1: Adams, Arapahoe, Douglas, Boulder, Denver, Broomfield and Jefferson counties; Region 2: Larimer, Weld and Routt counties; Region 3: El Paso and Teller counties

© 2022 Schrag SJ et al. *JAMA Network Open.*

eTable 4. Characteristics of Hospitalizations Among Pregnant People With COVID-19–Like Illness[a] by COVID-19 mRNA Vaccination Status (Regardless of Pregnancy Status at Time of Vaccination) and SARS-CoV-2 Test Result, 10 States, June 1, 2021, to June 2, 2022[b]

Characteristic	Total, No. (Column %)	No. (Row %)					SMD**	No. (Row %)	SMD**
		mRNA COVID-19 Vaccination Status						Positive SARS-CoV-2 Test Result	
		Unvaccinated	2 doses (14-149 days earlier)	2 doses (≥150 days earlier)	3 doses[¶] (7-119 days earlier)	3 doses[¶] (≥120 days earlier)			
**All Hospitalizations**	1073	670	123	169	84	27		351	
**Pregnancy trimester of CLI event**									
1st	39 (3.6)	30 (76.9)	2 (5.1)	3 (7.7)	3 (7.7)	1 (2.6)	0.29	18 (46.2)	0.23
2nd	183 (17.)	135 (73.8)	12 (6.6)	26 (14.2)	7 (3.8)	3 (1.6)		77 (42.1)	
3rd	851 (79.3)	505 (59.3)	109 (12.8)	140 (16.5)	74 (8.7)	23 (2.7)		256 (30.1)	
**CLI event associated with delivery**							0.25		0.49
No	378 (35.2)	263 (69.6)	34 (9.0)	47 (12.4)	21 (5.6)	13 (3.4)		178 (47.1)	
Yes	695 (64.8)	407 (58.6)	89 (12.8)	122 (17.6)	63 (9.1)	14 (2.0)		173 (24.9)	
**Variant predominance period**									
B.1.617.2 (Delta)	706 (65.8)	498 (70.5)	102 (14.4)	83 (11.8)	23 (3.3)	0 (0.0)	1.06	258 (36.5)	0.25
B.1.1.529 (Omicron)	367 (34.2)	172 (46.9)	21 (5.7)	86 (23.4)	61 (16.6)	27 (7.4)		93 (25.3)	
**Sites**									
Baylor Scott & White Health	39 (3.6)	27 (69.2)	5 (12.8)	6 (15.4)	1 (2.6)	0 (0.0)	0.71	8 (20.5)	0.40
Columbia University	90 (8.4)	50 (55.6)	13 (14.4)	22 (24.4)	4 (4.4)	1 (1.1)		17 (18.9)	
HealthPartners	19 (1.8)	11 (57.9)	1 (5.3)	4 (21.1)	2 (10.5)	1 (5.3)		5 (26.3)	
Intermountain Healthcare	115 (10.7)	83 (72.2)	7 (6.1)	19 (16.5)	5 (4.3)	1 (0.9)		27 (23.5)	
Kaiser Permanente Northern California	385 (35.9)	192 (49.9)	55 (14.3)	76 (19.7)	47 (12.2)	15 (3.9)		142 (36.9)	
Kaiser Permanente Northwest	73 (6.8)	38 (52.1)	9 (12.3)	9 (12.3)	12 (16.4)	5 (6.8)		20 (27.4)	
Regenstrief Institute	183 (17.2)	147 (80.3)	16 (8.7)	9 (4.9)	8 (4.4)	3 (1.6)		85 (46.4)	
University of Colorado	169 (15.8)	122 (72.2)	17 (10.1)	24 (14.2)	5 (3.0)	1 (0.6)		47 (27.8)	
**Age (yrs)**									
18-24	179 (16.7)	143 (79.9)	18 (10.1)	15 (8.4)	3 (1.7)	0 (0.0)	0.62	54 (30.2)	0.10
25-34	577 (53.8)	379 (65.7)	64 (11.1)	86 (14.9)	35 (6.1)	13 (2.3)		200 (34.7)	
35-45	317 (29.5)	148 (46.7)	41 (12.9)	68 (21.5)	46 (14.5)	14 (4.4)		97 (30.6)	
**Race/ethnicity**									

© 2022 Schrag SJ et al. *JAMA Network Open.*

Hispanic	269 (25.1)	167 (62.1)	31 (11.5)	51 (19.0)	15 (5.6)	5 (1.9)	0.40	80 (29.7)	0.26
Non-Hispanic Black	130 (12.1)	97 (74.6)	10 (7.7)	15 (11.5)	8 (6.2)	0 (0.0)		63 (48.5)	
Non-Hispanic Other[§§]	162 (15.1)	80 (49.4)	23 (14.2)	33 (20.4)	20 (12.3)	6 (3.7)		51 (31.5)	
Non-Hispanic White	473 (44.1)	305 (64.5)	53 (11.2)	64 (13.5)	36 (7.6)	15 (3.2)		145 (30.7)	
Unknown	39 (3.6)	21 (53.8)	6 (15.4)	6 (15.4)	5 (12.8)	1 (2.6)		12 (30.8)	
**Chronic respiratory condition[¶¶]**									
No	567 (52.8)	316 (55.7)	78 (13.8)	103 (18.2)	53 (9.3)	17 (3.0)	0.31	135 (27.1)	0.43
Yes	506 (47.2)	354 (70.0)	45 (8.9)	66 (13.0)	31 (6.1)	10 (2.0)		216 (37.6)	
**Chronic non respiratory condition[***]**									
No	498 (46.4)	308 (61.8)	52 (10.4)	91 (18.3)	34 (6.8)	13 (2.6)	0.10	812 (19.5)	0.24
Yes	575 (53.6)	362 (63.0)	71 (12.3)	78 (13.6)	50 (8.7)	14 (2.4)		73 (20.2)	
**ICU**									
No	938 (87.4)	574 (61.2)	110 (11.7)	152 (16.2)	76 (8.1)	26 (2.8)	0.19	291 (31.0)	0.20
Yes	135 (12.6)	96 (71.1)	13 (9.6)	17 (12.6)	8 (5.9)	1 (0.7)		60 (44.4)	
**Immunocompromised status**									
No	1034 (96.4)	648 (62.7)	116 (11.2)	163 (15.8)	80 (7.7)	27 (2.6)	0.12	342 (33.1)	0.09
Yes	39 (3.6)	22 (56.4)	7 (17.9)	6 (15.4)	4 (10.3)	0 (0.0)		9 (23.1)	
**History of SARSCoV-2 infection**									
No	976 (91.0)	612 (62.7)	111 (11.4)	152 (15.6)	76 (7.8)	25 (2.6)	0.04	345 (35.3)	0.43
Yes	97 (9.0)	58 (59.8)	12 (12.4)	17 (17.5)	8 (8.2)	2 (2.1)		6 (6.2)	
**Total vaccinated**	**403**	**0**	**123**	**169**	**84**	**65**		**27**	
**Vaccine product**									
Combination of mRNA products	7 (1.7)	0 (0.0)	0 (0.0)	0 (0.0)	6 (85.7)	1 (14.3)	NA	1 (14.3)	0.11
Moderna	144 (35.7)	0 (0.0)	45 (31.2)	63 (43.8)	25 (17.4)	11 (7.6)		12 (8.3)	
Pfizer-BioNTech	252 (62.5)	0 (0.0)	78 (31.0)	106 (42.1)	53 (21.0)	15 (6.0)		25 (9.9)	
**Timing of first dose**	**403**								
before current pregnancy	216 (53.6)	0 (0.0)	4 (1.9)	114 (52.8)	71 (32.9)	27 (12.5)	NA	28 (13.0)	0.59
1st trimester	84 (20.8)	0 (0.0)	22 (26.2)	49 (58.3)	13 (15.5)	0 (0.0)		7 (8.3)	
2nd trimester	84 (20.8)	0 (0.0)	78 (92.9)	6 (7.1)	0 (0.0)	0 (0.0)		3 (3.6)	
3rd trimester	19 (4.7)	0 (0.0)	19 (100.0)	0 (0.0)	0 (0.0)	0 (0.0)		0 (0.0)	
**Timing of second dose**	**403**								
before current pregnancy	182 (45.2)	0 (0.0)	2 (1.1)	91 (50.0)	62 (34.1)	27 (14.8)	NA	25 (13.7)	0.64
1st trimester	90 (22.3)	0 (0.0)	9 (10.0)	59 (65.6)	22 (24.4)	0 (0.0)		8 (8.9)	

© 2022 Schrag SJ et al. *JAMA Network Open.*

2nd trimester	94 (23.2)	0 (0.0)	75 (79.8)	19 (20.2)	0 (0.0)	0 (0.0)		5 (5.3)	
3rd trimester	37 (9.2)	0 (0.0)	37 (100.0)	0 (0.0)	0 (0.0)	0 (0.0)		0 (0.0)	
**Timing of third dose**	**292**								
1st trimester	10 (3.4)	0 (0.0)	0 (0.0)	0 (0.0)	2 (20.0)	8 (80.0)	NA	2 (20.0)	0.46
2nd trimester	52 (17.8)	0 (0.0)	0 (0.0)	0 (0.0)	36 (69.2)	16 (30.8)		4 (7.7)	
3rd trimester	44 (15.1)	0 (0.0)	0 (0.0)	0 (0.0)	44 (100.0)	0 (0.0)		3 (6.8)	
**2 Doses**									
**2 doses during current pregnancy**									
No	118 (40.4)	0 (0.0)	4 (3.4)	114 (96.6)	0 (0.0)	0 (0.0)	NA	18 (15.3)	0.55
Yes	174 (59.6)	0 (0.0)	119 (68.4)	55 (31.6)	0 (0.0)	0 (0.0)		10 (5.7)	
**Timing of first dose**	**Timing of 2nd dose**								
before current pregnancy	before current pregnancy		2	91	NA		NA	16	NA
Before current pregnancy	1st trimester		1	23				2	
before current pregnancy	2nd trimester		1	0				0	
1st trimester	1st trimester		8	36				5	
1st trimester	2nd trimester		13	13				2	
1st trimester	3rd trimester		1	0				0	
2nd trimester	2nd trimester		61	6				3	
2nd trimester	3rd trimester		17	0				0	
3rd trimester	3rd trimester		19	0				0	
**3 Doses**									
**3 doses during current pregnancy**									
No	98 (88.3)	0 (0.0)	0 (0.0)	0 (0.0)	71 (72.4)	27 (27.6)	NA	10 (10.2)	0.54
Yes	13 (11.7)	0 (0.0)	0 (0.0)	0 (0.0)	13 (100.0)	0 (0.0)		0 (0.0)	
**Timing of first dose**	**Timing of 2nd dose**	**Timing of 3rd dose**							
before current pregnancy	before current pregnancy	before current pregnancy			2	3	NA	1	NA
before current pregnancy	before current pregnancy	1st trimester	NA		2	8		2	
before current pregnancy	before current pregnancy	2nd trimester			34	16		4	
before current pregnancy	before current pregnancy	3rd trimester			24	0		2	
before current pregnancy	1st trimester	2nd trimester			1	0		0	

© 2022 Schrag SJ et al. *JAMA Network Open.*

before current pregnancy	1st trimester	3rd trimester		8	0		1	
1st trimester	1st trimester	2nd trimester		1	0		0	
1st trimester	1st trimester	3rd trimester		12	0		0	

Abbreviations: ICD-9 = International Classification of Diseases, Ninth Revision; ICD-10 = International Classification of Diseases, Tenth Revision; SMD = standardized mean or proportion difference.

[a]Medical events with an encounter or discharge code consistent with COVID-19–like illness were included, using ICD-9 and ICD-10. Four categories of codes were considered: 1) acute respiratory illness, including respiratory failure, viral or bacterial pneumonia, asthma exacerbation, influenza, and viral illness not otherwise specified; 2) non-respiratory COVID-19–like illness diagnoses including cause-unspecified gastroenteritis, thrombosis, and acute myocarditis; 3) respiratory signs and symptoms consistent with COVID-19–like illness, including hemoptysis, cough, dyspnea, painful respiration, or hypoxemia; 4) signs and symptoms of acute febrile illness. One code in any of the four categories was sufficient for inclusion. Clinician-ordered molecular assays (e.g., real-time reverse transcription–polymerase chain reaction) for SARS-CoV-2 occurring ≤14 days before to <72 hours after the encounter date were included.

[b]Partners contributing data on medical events were in California (estimated start date of Delta predominance June 23, estimated start date of Omicron predominance: December 21), Colorado (estimated start date of Delta predominance June 3, estimated start date of Omicron predominance December 19), Indiana (estimated start date of Delta predominance July 3, estimated start date of Omicron predominance December 26), Minnesota and Wisconsin (estimated start date of Delta predominance July 1, estimated start date of Omicron predominance December 25), New York (estimated start date of Delta predominance June 30, estimated start date of Omicron predominance December 18), Oregon (estimated start date of Delta predominance June 30, estimated start date of Omicron predominance December 24), Texas (estimated start date of Delta predominance September 11, estimated start date of Omicron predominance December 16), Utah (estimated start date of Delta predominance June 1, estimated start date of Omicron predominance December 24), and Washington (estimated star date Delta predominance June 30, estimated start date of Omicron predominance December 24). The study period began in September 2021 for partners located in Texas.

[c] An absolute standardized mean or proportion difference (SMD) ≥0.20 indicates a non-negligible difference in the distribution of characteristics for vaccinated categories versus unvaccinated patients and for positive versus negative test results. When calculating SMDs for differences in characteristics across COVID-19 vaccination status, investigators calculated SMD as the average of the three absolute values of the SMD for unvaccinated versus each vaccination status category individually (unvaccinated versus 2 doses 14-149 days earlier, unvaccinated versus 2-dose >=150 days earlier, and unvaccinated versus 3 mRNA doses ≥7 days earlier). All SMDs are reported as the absolute SMD. Additionally, the average SMD calculation comparing Negative SARS-CoV-2 Test Result and Positive SARS-CoV-2 Test Result was generated by directly calculating the SMD for Negative SARS-CoV-2 Test Result and Positive SARS-CoV-2 Test Result.

[d] Vaccination was defined as having received the listed number of doses of COVID-19 Pfizer-BioNTech BNT162b2 or Moderna mRNA-1273 vaccine ≥14 days (for 2 doses) or ≥7 days (for 3 doses) before the medical event index date, which was the date of respiratory specimen collection associated with the most recent positive or negative SARS-CoV-2 test result before medical event or the admission date if testing only occurred after the admission.

[e]Unknown race/ethnicity includes Asian, Native Hawaiian or other Pacific islander, American Indian or Alaska Native, other not listed, and multiple races.

[f]Chronic respiratory condition was defined as the presence of discharge code for asthma, COPD, or other lung disease using diagnosis codes from the ICD-9 and ICD-10

[g]Chronic non-respiratory condition was defined as the presence of discharge code for heart failure, ischemic heart disease, hypertension, other heart disease, stroke, other cerebrovascular disease, diabetes type I or II, other diabetes, metabolic disease, clinical obesity, clinically underweight, renal disease, liver disease, blood disorder, immunosuppression, organ transplant, cancer, neurological disorder, musculoskeletal disorder, Down Syndrome, and dementia

© 2022 Schrag SJ et al. *JAMA Network Open.*

eTable 5. Characteristics of Emergency Department and Urgent Care Encounters Among Pregnant People With COVID-19–Like Illness[a] by COVID-19 mRNA Vaccination Status (Regardless of Pregnancy Status at Time of Vaccination) and SARS-CoV-2 Test Result, 10 States, June 1, 2021, to June 2, 2022[b]

Characteristic	Total, No. (Column %)	No. (Row %)					SMD**	No. (Row %)	SMD**
		mRNA COVID-19 Vaccination Status						Positive SARS-CoV-2 Test Result	
		Unvaccinated	2 doses (14-149 days earlier)	2 doses (≥150 days earlier)	3 doses¶ (7-119 days earlier)	3 doses¶ (≥120 days earlier)			
**All ED/UC CLI encounters**	6196	3380	862	1366	433	155		1141	
**Pregnancy trimester of CLI event**									
1st	2165 (34.9)	1095 (50.6)	363 (16.8)	515 (23.8)	139 (6.4)	53 (2.4)	0.12	347 (16.0)	0.12
2nd	2200 (35.5)	1208 (54.9)	275 (12.5)	505 (23.0)	152 (6.9)	60 (2.7)		436 (19.8)	
3rd	1831 (29.6)	1077 (58.8)	224 (12.2)	346 (18.9)	142 (7.8)	42 (2.3)		358 (19.6)	
**CLI event associated with delivery**									
No	5725 (92.4)	3107 (54.3)	808 (14.1)	1258 (22.0)	406 (7.1)	146 (2.6)	0.06	1057 (18.5)	0.01
Yes	471 (7.6)	273 (58.0)	54 (11.5)	108 (22.9)	27 (5.7)	9 (1.9)		84 (17.8)	
**Variant predominance period**									
B.1.617.2 (Delta)	3635 (58.7)	2282 (62.8)	623 (17.1)	636 (17.5)	91 (2.5)	3 (0.1)	0.87	503 (13.8)	0.36
B.1.1.529 (Omicron)	2561 (41.3)	1098 (42.9)	239 (9.3)	730 (28.5)	342 (13.4)	152 (5.9)		638 (24.9)	
**Sites**									
Baylor Scott & White Health	111 (1.8)	73 (65.8)	14 (12.6)	21 (18.9)	2 (1.8)	1 (0.9)	0.63	42 (37.8)	0.33
Columbia University	294 (4.7)	192 (65.3)	50 (17.0)	38 (12.9)	13 (4.4)	1 (0.3)		40 (13.6)	
HealthPartners	433 (7.0)	183 (42.3)	65 (15.0)	115 (26.6)	45 (10.4)	25 (5.8)		92 (21.2)	
Intermountain Healthcare	2095 (33.8)	1213 (57.9)	281 (13.4)	439 (21.0)	122 (5.8)	40 (1.9)		288 (13.7)	
Kaiser Permanente Northern California	1561 (25.2)	636 (40.7)	285 (18.3)	434 (27.8)	156 (10.0)	50 (3.2)		275 (17.6)	
Kaiser Permanente Northwest	535 (8.6)	223 (41.7)	72 (13.5)	156 (29.2)	59 (11.0)	25 (4.7)		151 (28.2)	
Regenstrief Institute	690 (11.1)	550 (79.7)	48 (7.0)	70 (10.1)	18 (2.6)	4 (0.6)		151 (21.9)	
University of Colorado	477 (7.7)	310 (65.0)	47 (9.9)	93 (19.5)	18 (3.8)	9 (1.9)		102 (21.4)	
**Age (yrs)**									
18-24	1606 (25.9)	1139 (70.9)	178 (11.1)	241 (15.0)	35 (2.2)	13 (0.8)	0.57	280 (17.4)	0.04
25-34	3527 (56.9)	1833 (52.0)	493 (14.0)	847 (24.0)	265 (7.5)	89 (2.5)		660 (18.7)	
35-45	1063 (17.1)	408 (38.4)	191 (18.0)	278 (26.2)	133 (12.5)	53 (5.0)		201 (18.9)	
**Race/ethnicity**									

© 2022 Schrag SJ et al. *JAMA Network Open.*

Hispanic	1637 (26.4)	869 (53.1)	262 (16.0)	377 (23.0)	107 (6.5)	22 (1.3)	0.38	299 (18.3)	0.12
Non-Hispanic Black	690 (11.1)	464 (67.2)	80 (11.6)	114 (16.5)	23 (3.3)	9 (1.3)		163 (23.6)	
Non-Hispanic Other[§§]	604 (9.7)	220 (36.4)	95 (15.7)	189 (31.3)	71 (11.8)	29 (4.8)		100 (16.6)	
Non-Hispanic White	3080 (49.7)	1702 (55.3)	410 (13.3)	655 (21.3)	223 (7.2)	90 (2.9)		543 (17.6)	
Unknown	185 (3.0)	125 (67.6)	15 (8.1)	31 (16.8)	9 (4.9)	5 (2.7)		36 (19.5)	
**Chronic respiratory condition**[¶¶]									
No	5773 (93.2)	3116 (54.0)	818 (14.2)	1287 (22.3)	402 (7.0)	150 (2.6)	0.10	1057 (18.3)	0.03
Yes	423 (6.8)	264 (62.4)	44 (10.4)	79 (18.7)	31 (7.3)	5 (1.2)		84 (19.9)	
**Chronic non respiratory condition**[***]									
No	5727 (92.4)	3098 (54.1)	812 (14.2)	1272 (22.2)	402 (7.0)	143 (2.5)	0.06	1051 (18.4)	0.02
Yes	469 (7.6)	282 (60.1)	50 (10.7)	94 (20.0)	31 (6.6)	12 (2.6)		90 (19.2)	
**ICU**									
No	6164 (99.5)	3356 (54.4)	862 (14.0)	1361 (22.1)	431 (7.0)	154 (2.5)	0.05	1133 (18.4)	0.03
Yes	32 (0.5)	24 (75.0)	0 (0.0)	5 (15.6)	2 (6.2)	1 (3.1)		8 (25.0)	
**Immunocompromised status**									
No	6166 (99.5)	3362 (54.5)	859 (13.9)	1360 (22.1)	430 (7.0)	155 (2.5)	0.04	1136 (18.4)	0.01
Yes	30 (0.5)	18 (60.0)	3 (10.0)	6 (20.0)	3 (10.0)	0 (0.0)		5 (16.7)	
**History of SARSCoV-2 infection**									
No	5361 (86.5)	2940 (54.8)	749 (14.0)	1168 (21.8)	379 (7.1)	125 (2.3)	0.06	1066 (19.9)	0.28
Yes	835 (13.5)	440 (52.7)	113 (13.5)	198 (23.7)	54 (6.5)	30 (3.6)		75 (9.0)	
**Total vaccinated**	**2816**	**0**	**862**	**1366**	**433**	**155**		**373**	
**Vaccine product**									
Combination of mRNA products	71 (2.5)	0 (0.0)	1 (1.4)	3 (4.2)	51 (71.8)	16 (22.5)	NA	5 (7.0)	0.17
Moderna	899 (31.9)	0 (0.0)	244 (27.1)	490 (54.5)	126 (14.0)	39 (4.3)		100 (11.1)	
Pfizer-BioNTech	1846 (65.6)	0 (0.0)	617 (33.4)	873 (47.3)	256 (13.9)	100 (5.4)		268 (14.5)	
**Timing of first dose**	**2816**								
before current pregnancy	2268 (80.5)	0 (0.0)	436 (19.2)	1255 (55.3)	422 (18.6)	155 (6.8)	NA	316 (13.9)	0.17
1st trimester	341 (12.1)	0 (0.0)	224 (65.7)	106 (31.1)	11 (3.2)	0 (0.0)		34 (10.0)	
2nd trimester	192 (6.8)	0 (0.0)	187 (97.4)	5 (2.6)	0 (0.0)	0 (0.0)		23 (12.0)	
3rd trimester	15 (0.5)	0 (0.0)	15 (100.0)	0 (0.0)	0 (0.0)	0 (0.0)		0 (0.0)	
**Timing of second dose**	**2816**								
before current pregnancy	2074 (73.7)	0 (0.0)	339 (16.3)	1168 (56.3)	412 (19.9)	155 (7.5)	NA	291 (14.0)	0.18
1st trimester	465 (16.5)	0 (0.0)	263 (56.6)	181 (38.9)	21 (4.5)	0 (0.0)		55 (11.8)	

© 2022 Schrag SJ et al. *JAMA Network Open.*

2nd trimester	234 (8.3)	0 (0.0)	217 (92.7)	17 (7.3)	0 (0.0)	0 (0.0)		26 (11.1)	
3rd trimester	43 (1.5)	0 (0.0)	43 (100.0)	0 (0.0)	0 (0.0)	0 (0.0)		1 (2.3)	
**Timing of third dose**	**588**								
1st trimester	180 (30.6)	0 (0.0)	0 (0.0)	0 (0.0)	134 (74.4)	46 (25.6)		18 (10.0)	
2nd trimester	186 (31.6)	0 (0.0)	0 (0.0)	0 (0.0)	167 (89.8)	19 (10.2)	NA	14 (7.5)	0.20
3rd trimester	50 (8.5)	0 (0.0)	0 (0.0)	0 (0.0)	50 (100.0)	0 (0.0)		4 (8.0)	
**2 Doses**									
**2 doses during current pregnancy**									
No	1691 (75.9)	0 (0.0)	436 (25.8)	1255 (74.2)	0 (0.0)	0 (0.0)	NA	260 (15.4)	0.17
Yes	537 (24.1)	0 (0.0)	426 (79.3)	111 (20.7)	0 (0.0)	0 (0.0)		57 (10.6)	
**Timing of first dose**	**Timing of 2nd dose**								
before current pregnancy	before current pregnancy		339	1168				236	
before current pregnancy	1st trimester		95	87				24	
before current pregnancy	2nd trimester		2	0				0	
1st trimester	1st trimester		168	94				30	
1st trimester	2nd trimester		54	12	NA		NA	4	NA
1st trimester	3rd trimester		2	0				0	
2nd trimester	2nd trimester		161	5				22	
2nd trimester	3rd trimester		26	0				1	
3rd trimester	3rd trimester		15	0				0	
**3 Doses**									
**3 doses during current pregnancy**									
No	577 (100.0)	0 (0.0)	0 (0.0)	0 (0.0)	422 (73.1)	155 (26.9)	NA	56 (9.7)	0.21
Yes	11 (100.0)	0 (0.0)	0 (0.0)	0 (0.0)	11 (100.0)	0 (0.0)		0 (0.0)	
**Timing of first dose**	**Timing of 2nd dose**	**Timing of 3rd dose**							
before current pregnancy	before current pregnancy	before current pregnancy			82	90		20	
before current pregnancy	before current pregnancy	1st trimester	NA		134	46		18	NA
before current pregnancy	before current pregnancy	2nd trimester			166	19		14	

© 2022 Schrag SJ et al. *JAMA Network Open.*

before current pregnancy	before current pregnancy	3rd trimester		30	0		0	
before current pregnancy	1st trimester	2nd trimester		1	0		0	
before current pregnancy	1st trimester	3rd trimester		9	0		3	
1st trimester	1st trimester	3rd trimester		11	0		1	

Abbreviations: ICD-9 = International Classification of Diseases, Ninth Revision; ICD-10 = International Classification of Diseases, Tenth Revision; SMD = standardized mean or proportion difference.

[a]Medical events with an encounter or discharge code consistent with COVID-19–like illness were included, using ICD-9 and ICD-10. Four categories of codes were considered: 1) acute respiratory illness, including respiratory failure, viral or bacterial pneumonia, asthma exacerbation, influenza, and viral illness not otherwise specified; 2) non-respiratory COVID-19–like illness diagnoses including cause-unspecified gastroenteritis, thrombosis, and acute myocarditis; 3) respiratory signs and symptoms consistent with COVID-19–like illness, including hemoptysis, cough, dyspnea, painful respiration, or hypoxemia; 4) signs and symptoms of acute febrile illness. One code in any of the four categories was sufficient for inclusion. Clinician-ordered molecular assays (e.g., real-time reverse transcription–polymerase chain reaction) for SARS-CoV-2 occurring ≤14 days before to <72 hours after the encounter date were included.

[b]Partners contributing data on medical events were in California (estimated start date of Delta predominance June 23, estimated start date of Omicron predominance: December 21), Colorado (estimated start date of Delta predominance June 3, estimated start date of Omicron predominance December 19), Indiana (estimated start date of Delta predominance July 3, estimated start date of Omicron predominance December 26), Minnesota and Wisconsin (estimated start date of Delta predominance July 1, estimated start date of Omicron predominance December 25), New York (estimated start date of Delta predominance June 30, estimated start date of Omicron predominance December 18), Oregon (estimated start date of Delta predominance June 30, estimated start date of Omicron predominance December 24), Texas (estimated start date of Delta predominance September 11, estimated start date of Omicron predominance December 16), Utah (estimated start date of Delta predominance June 1, estimated start date of Omicron predominance December 24), and Washington (estimated star date Delta predominance June 30, estimated start date of Omicron predominance December 24). The study period began in September 2021 for partners located in Texas.

[c] An absolute standardized mean or proportion difference (SMD) ≥0.20 indicates a non-negligible difference in the distribution of characteristics for vaccinated categories versus unvaccinated patients and for positive versus negative test results. When calculating SMDs for differences in characteristics across COVID-19 vaccination status, investigators calculated SMD as the average of the three absolute values of the SMD for unvaccinated versus each vaccination status category individually (unvaccinated versus 2 doses 14-149 days earlier, unvaccinated versus 2-dose >=150 days earlier, and unvaccinated versus 3 mRNA doses ≥7 days earlier). All SMDs are reported as the absolute SMD. Additionally, the average SMD calculation comparing Negative SARS-CoV-2 Test Result and Positive SARS-CoV-2 Test Result was generated by directly calculating the SMD for Negative SARS-CoV-2 Test Result and Positive SARS-CoV-2 Test Result.

[d] Vaccination was defined as having received the listed number of doses of COVID-19 Pfizer-BioNTech BNT162b2 or Moderna mRNA-1273 vaccine ≥14 days (for 2 doses) or ≥7 days (for 3 doses) before the medical event index date, which was the date of respiratory specimen collection associated with the most recent positive or negative SARS-CoV-2 test result before medical event or the admission date if testing only occurred after the admission.

[e]Unknown race/ethnicity includes Asian, Native Hawaiian or other Pacific islander, American Indian or Alaska Native, other not listed, and multiple races.

[f]Chronic respiratory condition was defined as the presence of discharge code for asthma, COPD, or other lung disease using diagnosis codes from the ICD-9 and ICD-10

[g]Chronic non-respiratory condition was defined as the presence of discharge code for heart failure, ischemic heart disease, hypertension, other heart disease, stroke, other cerebrovascular disease, diabetes type I or II, other diabetes, metabolic disease, clinical obesity, clinically underweight, renal disease, liver disease, blood disorder, immunosuppression, organ transplant, cancer, neurological disorder, musculoskeletal disorder, Down Syndrome, and dementia

© 2022 Schrag SJ et al. *JAMA Network Open.*

eTable 6. Characteristics of COVID-19–Like Illness Hospitalizations Among Nonpregnant Women Aged 18 to 45 Years With COVID-19–Like Illness[a] by COVID-19 BNT162b2 Vaccination Status and SARS-CoV-2 Test Result, 10 States, June 1, 2021, to June 2, 2022[b]

Characteristic	Total, No. (Column %)	No. (Row %) mRNA COVID-19 Vaccination Status					SMD**	No. (Row %)	SMD**
		Unvaccinated	2 doses (14-149 days earlier)	2 doses (≥150 days earlier)	3 doses[¶] (7-119 days earlier)	3 doses[¶] (≥120 days earlier)		Positive SARS-CoV-2 Test Result	
**All CLI Hospitalizations**	9297	6011	955	1563	549	219		2849	
**Variant predominance period**									
B.1.617.2 (Delta)	5703 (61.3)	4095 (71.8)	747 (13.1)	728 (12.8)	130 (2.3)	3 (0.1)	0.91	2067 (36.2)	0.34
B.1.1.529 (Omicron)	3594 (38.7)	1916 (53.3)	208 (5.8)	835 (23.2)	419 (11.7)	216 (6.0)		782 (21.8)	
**Sites**									
Baylor Scott & White Health	1916 (20.6)	1279 (66.8)	127 (6.6)	385 (20.1)	80 (4.2)	45 (2.3)	0.55	463 (24.2)	0.43
Columbia University	665 (7.2)	318 (47.8)	120 (18.0)	155 (23.3)	53 (8.0)	19 (2.9)		100 (15.0)	
HealthPartners	415 (4.5)	236 (56.9)	49 (11.8)	70 (16.9)	40 (9.6)	20 (4.8)		118 (28.4)	
Intermountain Healthcare	1096 (11.8)	688 (62.8)	117 (10.7)	192 (17.5)	72 (6.6)	27 (2.5)		299 (27.3)	
Kaiser Permanente Northern California	1454 (15.6)	764 (52.5)	194 (13.3)	272 (18.7)	159 (10.9)	65 (4.5)		667 (45.9)	
Kaiser Permanente Northwest	338 (3.6)	184 (54.4)	38 (11.2)	73 (21.6)	33 (9.8)	10 (3.0)		90 (26.6)	
Regenstrief Institute	2301 (24.7)	1778 (77.3)	174 (7.6)	258 (11.2)	72 (3.1)	19 (0.8)		848 (36.9)	
University of Colorado	1112 (12.0)	764 (68.7)	136 (12.2)	158 (14.2)	40 (3.6)	14 (1.3)		264 (23.7)	
**Age (yrs)**									
18-24	1264 (13.6)	840 (66.5)	142 (11.2)	198 (15.7)	66 (5.2)	18 (1.4)	0.15	257 (20.3)	0.21
25-34	2883 (31.0)	1951 (67.7)	281 (9.7)	459 (15.9)	130 (4.5)	62 (2.2)		881 (30.6)	
35-45	5150 (55.4)	3220 (62.5)	532 (10.3)	906 (17.6)	353 (6.9)	139 (2.7)		1711 (33.2)	
**Race/ethnicity**									
Hispanic	1909 (20.5)	1200 (62.9)	220 (11.5)	338 (17.7)	112 (5.9)	39 (2.0)	0.23	692 (36.2)	0.15
Non-Hispanic Black	1756 (18.9)	1231 (70.1)	160 (9.1)	260 (14.8)	73 (4.2)	32 (1.8)		503 (28.6)	
Non-Hispanic Other[§§]	803 (8.6)	428 (53.3)	114 (14.2)	161 (20.0)	72 (9.0)	28 (3.5)		250 (31.1)	
Non-Hispanic White	4441 (47.8)	2866 (64.5)	431 (9.7)	755 (17.0)	275 (6.2)	114 (2.6)		1264 (28.5)	
Unknown	388 (4.2)	286 (73.7)	30 (7.7)	49 (12.6)	17 (4.4)	6 (1.5)		140 (36.1)	
**Chronic respiratory condition[¶¶]**									
No	3353 (36.1)	2020 (60.2)	414 (12.3)	617 (18.4)	204 (6.1)	98 (2.9)	0.16	780 (23.3)	0.27
Yes	5944 (63.9)	3991 (67.1)	541 (9.1)	946 (15.9)	345 (5.8)	121 (2.0)		2069 (34.8)	

© 2022 Schrag SJ et al. *JAMA Network Open.*

**Chronic non respiratory condition*** **									
No	1992 (21.4)	1457 (73.1)	158 (7.9)	249 (12.5)	92 (4.6)	36 (1.8)	0.20	707 (35.5)	0.12
Yes	7305 (78.6)	4554 (62.3)	797 (10.9)	1314 (18.0)	457 (6.3)	183 (2.5)		2142 (29.3)	
**ICU**									
No	7415 (79.8)	4813 (64.9)	743 (10.0)	1235 (16.7)	444 (6.0)	180 (2.4)	0.04	2368 (31.9)	0.12
Yes	1882 (20.2)	1198 (63.7)	212 (11.3)	328 (17.4)	105 (5.6)	39 (2.1)		481 (25.6)	
**Immunocompromised status**									
No	7458 (80.2)	5100 (68.4)	692 (9.3)	1158 (15.5)	368 (4.9)	140 (1.9)	0.37	2506 (33.6)	0.16
Yes	1839 (19.8)	911 (49.5)	263 (14.3)	405 (22.0)	181 (9.8)	79 (4.3)		343 (18.7)	
**History of SARSCoV-2 infection**									
No	8316 (89.4)	5484 (65.9)	835 (10.0)	1338 (16.1)	471 (5.7)	188 (2.3)	0.16	2759 (33.2)	0.39
Yes	981 (10.6)	527 (53.7)	120 (12.2)	225 (22.9)	78 (8.0)	31 (3.2)		90 (9.2)	
**Total vaccinated**	**3286**	**0**	**955**	**1563**	**549**	**219**		**346**	
**Vaccine product**							NA		0.19
Combination of mRNA products	63 (1.9)	0 (0.0)	5 (7.9)	2 (3.2)	45 (71.4)	11 (17.5)		5 (7.9)	
Moderna	1178 (35.8)	0 (0.0)	335 (28.4)	573 (48.6)	195 (16.6)	75 (6.4)		98 (8.3)	
Pfizer-BioNTech	2045 (62.2)	0 (0.0)	615 (30.1)	988 (48.3)	309 (15.1)	133 (6.5)		243 (11.9)	

Abbreviations: ICD-9 = International Classification of Diseases, Ninth Revision; ICD-10 = International Classification of Diseases, Tenth Revision; SMD = standardized mean or proportion difference.

[a]Medical events with an encounter or discharge code consistent with COVID-19–like illness were included, using ICD-9 and ICD-10. Four categories of codes were considered: 1) acute respiratory illness, including respiratory failure, viral or bacterial pneumonia, asthma exacerbation, influenza, and viral illness not otherwise specified; 2) non-respiratory COVID-19–like illness diagnoses including cause-unspecified gastroenteritis, thrombosis, and acute myocarditis; 3) respiratory signs and symptoms consistent with COVID-19–like illness, including hemoptysis, cough, dyspnea, painful respiration, or hypoxemia; 4) signs and symptoms of acute febrile illness. One code in any of the four categories was sufficient for inclusion. Clinician-ordered molecular assays (e.g., real-time reverse transcription–polymerase chain reaction) for SARS-CoV-2 occurring ≤14 days before to <72 hours after the encounter date were included.

[b]Partners contributing data on medical events were in California (estimated start date of Delta predominance June 23, estimated start date of Omicron predominance: December 21), Colorado (estimated start date of Delta predominance June 3, estimated start date of Omicron predominance December 19), Indiana (estimated start date of Delta predominance July 3, estimated start date of Omicron predominance December 26), Minnesota and Wisconsin (estimated start date of Delta predominance July 1, estimated start date of Omicron predominance December 25), New York (estimated start date of Delta predominance June 30, estimated start date of Omicron predominance December 18), Oregon (estimated start date of Delta predominance June 30, estimated start date of Omicron predominance December 24), Texas (estimated start date of Delta predominance September 11, estimated start date of Omicron predominance December 16), Utah (estimated start date of Delta predominance June 1, estimated start date of Omicron predominance December 24), and Washington (estimated star date Delta predominance June 30, estimated start date of Omicron predominance December 24). The study period began in September 2021 for partners located in Texas.

© 2022 Schrag SJ et al. *JAMA Network Open.*

[c] An absolute standardized mean or proportion difference (SMD) ≥0.20 indicates a non-negligible difference in the distribution of characteristics for vaccinated categories versus unvaccinated patients and for positive versus negative test results. When calculating SMDs for differences in characteristics across COVID-19 vaccination status, investigators calculated SMD as the average of the three absolute values of the SMD for unvaccinated versus each vaccination status category individually (unvaccinated versus 2 doses 14-149 days earlier, unvaccinated versus 2-dose >=150 days earlier, and unvaccinated versus 3 mRNA doses ≥7 days earlier). All SMDs are reported as the absolute SMD. Additionally, the average SMD calculation comparing Negative SARS-CoV-2 Test Result and Positive SARS-CoV-2 Test Result was generated by directly calculating the SMD for Negative SARS-CoV-2 Test Result and Positive SARS-CoV-2 Test Result.

[d] Vaccination was defined as having received the listed number of doses of COVID-19 Pfizer-BioNTech BNT162b2 or Moderna mRNA-1273 vaccine ≥14 days (for 2 doses) or ≥7 days (for 3 doses) before the medical event index date, which was the date of respiratory specimen collection associated with the most recent positive or negative SARS-CoV-2 test result before medical event or the admission date if testing only occurred after the admission.

[e]Unknown race/ethnicity includes Asian, Native Hawaiian or other Pacific islander, American Indian or Alaska Native, other not listed, and multiple races.

[f]Chronic respiratory condition was defined as the presence of discharge code for asthma, COPD, or other lung disease using diagnosis codes from the ICD-9 and ICD-10

[g]Chronic non-respiratory condition was defined as the presence of discharge code for heart failure, ischemic heart disease, hypertension, other heart disease, stroke, other cerebrovascular disease, diabetes type I or II, other diabetes, metabolic disease, clinical obesity, clinically underweight, renal disease, liver disease, blood disorder, immunosuppression, organ transplant, cancer, neurological disorder, musculoskeletal disorder, Down Syndrome, and dementia

© 2022 Schrag SJ et al. *JAMA Network Open.*

eTable 7. Characteristics of Emergency Department and Urgent Care Encounters Among Nonpregnant Women Aged 18 to 45 Years With COVID-19–Like Illness[a] by COVID-19 BNT162b2 Vaccination Status and SARS-CoV-2 Test Result, 10 States, June 1, 2021, to June 2, 2022[b]

Characteristic	Total, No. (Column %)	No. (Row %)					SMD**	No. (Row %)	SMD**
		mRNA COVID-19 Vaccination Status						Positive SARS-CoV-2 Test Result	
		Unvaccinated	2 doses (14-149 days earlier)	2 doses (≥150 days earlier)	3 doses[¶] (7-119 days earlier)	3 doses[¶] (≥120 days earlier)			
**All ED/UC CLI Visits**	154289	77332	22400	37538	11982	5037		27413	
**Variant predominance period**									
B.1.617.2 (Delta)	85057 (55.1)	45588 (53.6)	17523 (20.6)	19280 (22.7)	2608 (3.1)	58 (0.1)	0.76	10991 (12.9)	0.37
B.1.1.529 (Omicron)	69232 (44.9)	31744 (45.9)	4877 (7.0)	18258 (26.4)	9374 (13.5)	4979 (7.2)		16422 (23.7)	
**Sites**									
Baylor Scott & White Health	21039 (13.6)	14652 (69.6)	1570 (7.5)	3964 (18.8)	585 (2.8)	268 (1.3)	0.72	5913 (28.1)	0.34
Columbia University	4278 (2.8)	2394 (56.0)	647 (15.1)	949 (22.2)	223 (5.2)	65 (1.5)		600 (14.0)	
HealthPartners	22232 (14.4)	8002 (36.0)	3676 (16.5)	6522 (29.3)	2653 (11.9)	1379 (6.2)		3819 (17.2)	
Intermountain Healthcare	41193 (26.7)	18572 (45.1)	6815 (16.5)	11192 (27.2)	3270 (7.9)	1344 (3.3)		5058 (12.3)	
Kaiser Permanente Northern California	18656 (12.1)	5879 (31.5)	3830 (20.5)	5253 (28.2)	2582 (13.8)	1112 (6.0)		2823 (15.1)	
Kaiser Permanente Northwest	9607 (6.2)	3683 (38.3)	1614 (16.8)	2715 (28.3)	1103 (11.5)	492 (5.1)		1780 (18.5)	
Regenstrief Institute	21512 (13.9)	14949 (69.5)	2333 (10.8)	3250 (15.1)	810 (3.8)	170 (0.8)		4621 (21.5)	
University of Colorado	15772 (10.2)	9201 (58.3)	1915 (12.1)	3693 (23.4)	756 (4.8)	207 (1.3)		2799 (17.7)	
**Age (yrs)**									
18-24	41987 (27.2)	22872 (54.5)	6426 (15.3)	9582 (22.8)	2317 (5.5)	790 (1.9)	0.24	6191 (14.7)	0.14
25-34	56587 (36.7)	29584 (52.3)	8058 (14.2)	13093 (23.1)	4137 (7.3)	1715 (3.0)		10155 (17.9)	
35-45	55715 (36.1)	24876 (44.6)	7916 (14.2)	14863 (26.7)	5528 (9.9)	2532 (4.5)		11067 (19.9)	
**Race/ethnicity**									
Hispanic	29237 (18.9)	14660 (50.1)	4515 (15.4)	7154 (24.5)	2131 (7.3)	777 (2.7)	0.33	5499 (18.8)	0.23
Non-Hispanic Black	19650 (12.7)	12739 (64.8)	2550 (13.0)	3367 (17.1)	713 (3.6)	281 (1.4)		4916 (25.0)	
Non-Hispanic Other[§§]	12506 (8.1)	4710 (37.7)	2138 (17.1)	3527 (28.2)	1459 (11.7)	672 (5.4)		2213 (17.7)	
Non-Hispanic White	86290 (55.9)	41396 (48.0)	12322 (14.3)	22140 (25.7)	7242 (8.4)	3190 (3.7)		13193 (15.3)	
Unknown	6606 (4.3)	3827 (57.9)	875 (13.2)	1350 (20.4)	437 (6.6)	117 (1.8)		1592 (24.1)	
**Chronic respiratory condition[¶¶]**									
No	139446 (90.4)	69499 (49.8)	20389 (14.6)	34098 (24.5)	10876 (7.8)	4584 (3.3)	0.04	24933 (17.9)	0.02
Yes	14843 (9.6)	7833 (52.8)	2011 (13.5)	3440 (23.2)	1106 (7.5)	453 (3.1)		2480 (16.7)	

© 2022 Schrag SJ et al. *JAMA Network Open.*

**Chronic non respiratory condition*** **									
No	137189 (88.9)	68118 (49.7)	20144 (14.7)	33790 (24.6)	10701 (7.8)	4436 (3.2)	0.04	24609 (17.9)	0.03
Yes	17100 (11.1)	9214 (53.9)	2256 (13.2)	3748 (21.9)	1281 (7.5)	601 (3.5)		2804 (16.4)	
**ICU**									
No	152696 (99.0)	76289 (50.0)	22241 (14.6)	37265 (24.4)	11910 (7.8)	4991 (3.3)	0.06	27097 (17.7)	0.01
Yes	1593 (1.0)	1043 (65.5)	159 (10.0)	273 (17.1)	72 (4.5)	46 (2.9)		316 (19.8)	
**Immunocompromised status**									
No	151927 (98.5)	76225 (50.2)	22109 (14.6)	36981 (24.3)	11731 (7.7)	4881 (3.2)	0.05	27073 (17.8)	0.03
Yes	2362 (1.5)	1107 (46.9)	291 (12.3)	557 (23.6)	251 (10.6)	156 (6.6)		340 (14.4)	
**History of SARSCoV-2 infection**									
No	134934 (87.5)	67700 (50.2)	19620 (14.5)	32810 (24.3)	10506 (7.8)	4298 (3.2)	0.02	25654 (19.0)	0.25
Yes	19355 (12.5)	9632 (49.8)	2780 (14.4)	4728 (24.4)	1476 (7.6)	739 (3.8)		1759 (9.1)	
**Total vaccinated**	**76957**	**0**	**22400**	**37538**	**11982**	**219**		**5037**	
**Vaccine product**									
Combination of mRNA products	2034 (2.6)	0 (0.0)	82 (4.0)	55 (2.7)	1365 (67.1)	532 (26.2)	NA	203 (10.0)	0.09
Moderna	26053 (33.9)	0 (0.0)	6888 (26.4)	14009 (53.8)	3697 (14.2)	1459 (5.6)		2699 (10.4)	
Pfizer-BioNTech	48870 (63.5)	0 (0.0)	15430 (31.6)	23474 (48.0)	6920 (14.2)	3046 (6.2)		5968 (12.2)	

Abbreviations: ICD-9 = International Classification of Diseases, Ninth Revision; ICD-10 = International Classification of Diseases, Tenth Revision; SMD = standardized mean or proportion difference.

[a]Medical events with an encounter or discharge code consistent with COVID-19–like illness were included, using ICD-9 and ICD-10. Four categories of codes were considered: 1) acute respiratory illness, including respiratory failure, viral or bacterial pneumonia, asthma exacerbation, influenza, and viral illness not otherwise specified; 2) non-respiratory COVID-19–like illness diagnoses including cause-unspecified gastroenteritis, thrombosis, and acute myocarditis; 3) respiratory signs and symptoms consistent with COVID-19–like illness, including hemoptysis, cough, dyspnea, painful respiration, or hypoxemia; 4) signs and symptoms of acute febrile illness. One code in any of the four categories was sufficient for inclusion. Clinician-ordered molecular assays (e.g., real-time reverse transcription–polymerase chain reaction) for SARS-CoV-2 occurring ≤14 days before to <72 hours after the encounter date were included.

[b]Partners contributing data on medical events were in California (estimated start date of Delta predominance June 23, estimated start date of Omicron predominance: December 21), Colorado (estimated start date of Delta predominance June 3, estimated start date of Omicron predominance December 19), Indiana (estimated start date of Delta predominance July 3, estimated start date of Omicron predominance December 26), Minnesota and Wisconsin (estimated start date of Delta predominance July 1, estimated start date of Omicron predominance December 25), New York (estimated start date of Delta predominance June 30, estimated start date of Omicron predominance December 18), Oregon (estimated start date of Delta predominance June 30, estimated start date of Omicron predominance December 24), Texas (estimated start date of Delta predominance September 11, estimated start date of Omicron predominance December 16), Utah (estimated start date of Delta predominance June 1, estimated start date of Omicron predominance December 24), and Washington (estimated star date Delta predominance June 30, estimated start date of Omicron predominance December 24). The study period began in September 2021 for partners located in Texas.

© 2022 Schrag SJ et al. *JAMA Network Open.*

[c] An absolute standardized mean or proportion difference (SMD) ≥0.20 indicates a non-negligible difference in the distribution of characteristics for vaccinated categories versus unvaccinated patients and for positive versus negative test results. When calculating SMDs for differences in characteristics across COVID-19 vaccination status, investigators calculated SMD as the average of the three absolute values of the SMD for unvaccinated versus each vaccination status category individually (unvaccinated versus 2 doses 14-149 days earlier, unvaccinated versus 2-dose >=150 days earlier, and unvaccinated versus 3 mRNA doses ≥7 days earlier). All SMDs are reported as the absolute SMD. Additionally, the average SMD calculation comparing Negative SARS-CoV-2 Test Result and Positive SARS-CoV-2 Test Result was generated by directly calculating the SMD for Negative SARS-CoV-2 Test Result and Positive SARS-CoV-2 Test Result.

[d] Vaccination was defined as having received the listed number of doses of COVID-19 Pfizer-BioNTech BNT162b2 or Moderna mRNA-1273 vaccine ≥14 days (for 2 doses) or ≥7 days (for 3 doses) before the medical event index date, which was the date of respiratory specimen collection associated with the most recent positive or negative SARS-CoV-2 test result before medical event or the admission date if testing only occurred after the admission.

[e]Unknown race/ethnicity includes Asian, Native Hawaiian or other Pacific islander, American Indian or Alaska Native, other not listed, and multiple races.

[f]Chronic respiratory condition was defined as the presence of discharge code for asthma, COPD, or other lung disease using diagnosis codes from the ICD-9 and ICD-10

[g]Chronic non-respiratory condition was defined as the presence of discharge code for heart failure, ischemic heart disease, hypertension, other heart disease, stroke, other cerebrovascular disease, diabetes type I or II, other diabetes, metabolic disease, clinical obesity, clinically underweight, renal disease, liver disease, blood disorder, immunosuppression, organ transplant, cancer, neurological disorder, musculoskeletal disorder, Down Syndrome, and dementia.

© 2022 Schrag SJ et al. *JAMA Network Open.*

eTable 8. Characteristics of Hospitalizations Among Pregnant People With COVID-19–Like Illness[a] Who Did Not Have a Prior Positive SARS-CoV-2 Infection by COVID-19 mRNA Vaccination Status, VISION Network, 10 states, June 1, 2021–June 2, 2022[b]

Characteristic	Total, No. (Column %)	No. (Row %) mRNA COVID-19 Vaccination Status: Unvaccinated	2 doses (14-149 days earlier)	2 doses (≥150 days earlier)	3 doses[¶] (7-119 days earlier)	3 doses[¶] (≥120 days earlier)	SMD**	No. (Row %) Positive SARS-CoV-2 Test Result	SMD**
**All Hospitalizations**	888	612	110	70	74	22		329	
**Pregnancy trimester of CLI event**									
1st	28 (3.2)	27 (96.4)	0 (0.0)	0 (0.0)	1 (3.6)	0 (0.0)	0.50	17 (60.7)	0.31
2nd	150 (16.9)	127 (84.7)	11 (7.3)	5 (3.3)	6 (4.0)	1 (0.7)		74 (49.3)	
3rd	710 (80)	458 (64.5)	99 (13.9)	65 (9.2)	67 (9.4)	21 (3.0)		238 (33.5)	
**CLI event associated with delivery**							0.36		0.57
No	306 (34.5)	243 (79.4)	27 (8.8)	10 (3.3)	16 (5.2)	10 (3.3)		168 (54.9)	
Yes	582 (65.5)	369 (63.4)	83 (14.3)	60 (10.3)	58 (10.0)	12 (2.1)		161 (27.7)	
**Variant predominance period**									
B.1.617.2 (Delta)	638 (71.8)	469 (73.5)	94 (14.7)	54 (8.5)	21 (3.3)	0 (0.0)	0.98	255 (40.0)	0.20
B.1.1.529 (Omicron)	250 (28.2)	143 (57.2)	16 (6.4)	16 (6.4)	53 (21.2)	22 (8.8)		74 (29.6)	
**Sites**									
Baylor Scott & White Health	32 (3.6)	25 (78.1)	5 (15.6)	1 (3.1)	1 (3.1)	0 (0.0)	0.72	8 (25.0)	0.46
Columbia University	78 (8.8)	48 (61.5)	13 (16.7)	12 (15.4)	4 (5.1)	1 (1.3)		16 (20.5)	
HealthPartners	16 (1.8)	10 (62.5)	1 (6.2)	2 (12.5)	2 (12.5)	1 (6.2)		3 (18.8)	
Intermountain Healthcare	89 (10)	71 (79.8)	7 (7.9)	5 (5.6)	5 (5.6)	1 (1.1)		27 (30.3)	
Kaiser Permanente Northern California	312 (35.1)	180 (57.7)	48 (15.4)	32 (10.3)	42 (13.5)	10 (3.2)		129 (41.3)	
Kaiser Permanente Northwest	63 (7.1)	36 (57.1)	8 (12.7)	5 (7.9)	9 (14.3)	5 (7.9)		20 (31.7)	
Regenstrief Institute	153 (17.2)	131 (85.6)	12 (7.8)	1 (0.7)	6 (3.9)	3 (2.0)		83 (54.2)	
University of Colorado	145 (16.3)	111 (76.6)	16 (11.0)	12 (8.3)	5 (3.4)	1 (0.7)		43 (29.7)	
**Age (yrs)**									
18-24	149 (16.8)	126 (84.6)	15 (10.1)	5 (3.4)	3 (2.0)	0 (0.0)	0.63	51 (34.2)	0.11
25-34	480 (54.1)	349 (72.7)	55 (11.5)	32 (6.7)	32 (6.7)	12 (2.5)		189 (39.4)	
35-45	259 (29.2)	137 (52.9)	40 (15.4)	33 (12.7)	39 (15.1)	10 (3.9)		89 (34.4)	
**Race/ethnicity**									

© 2022 Schrag SJ et al. *JAMA Network Open.*

Hispanic	218 (24.5)	156 (71.6)	26 (11.9)	18 (8.3)	15 (6.9)	3 (1.4)		76 (34.9)	
Non-Hispanic Black	109 (12.3)	91 (83.5)	7 (6.4)	4 (3.7)	7 (6.4)	0 (0.0)		59 (54.1)	
Non-Hispanic Other[§§]	131 (14.8)	72 (55.0)	22 (16.8)	13 (9.9)	19 (14.5)	5 (3.8)	0.47	46 (35.1)	0.27
Non-Hispanic White	398 (44.8)	275 (69.1)	49 (12.3)	32 (8.0)	29 (7.3)	13 (3.3)		137 (34.4)	
Unknown	32 (3.6)	18 (56.2)	6 (18.8)	3 (9.4)	4 (12.5)	1 (3.1)		11 (34.4)	
**Chronic respiratory condition[¶¶]**									
No	461 (51.9)	285 (61.8)	70 (15.2)	45 (9.8)	48 (10.4)	13 (2.8)	0.34	125 (27.1)	0.43
Yes	427 (48.1)	327 (76.6)	40 (9.4)	25 (5.9)	26 (6.1)	9 (2.1)		204 (47.8)	
**Chronic non respiratory condition[***]**									
No	403 (45.4)	281 (69.7)	44 (10.9)	37 (9.2)	31 (7.7)	10 (2.5)	0.09	125 (31.0)	0.24
Yes	485 (54.6)	331 (68.2)	66 (13.6)	33 (6.8)	43 (8.9)	12 (2.5)		204 (42.1)	
**ICU**									
No	773 (87)	522 (67.5)	98 (12.7)	64 (8.3)	68 (8.8)	21 (2.7)	0.27	271 (35.1)	0.22
Yes	115 (13)	90 (78.3)	12 (10.4)	6 (5.2)	6 (5.2)	1 (0.9)		58 (50.4)	
**Immunocompromised status**									
No	855 (96.3)	591 (69.1)	104 (12.2)	67 (7.8)	71 (8.3)	22 (2.6)	0.11	321 (37.5)	0.11
Yes	33 (3.7)	21 (63.6)	6 (18.2)	3 (9.1)	3 (9.1)	0 (0.0)		8 (24.2)	
**History of SARSCoV-2 infection**									
No	888 (100)	612 (68.9)	110 (12.4)	70 (7.9)	74 (8.3)	22 (2.5)	NA	329 (37.0)	NA
Yes	0 (0)	0 (0)	0 (0)	0 (0)	0 (0)	0 (0)		0(0)	
**Total vaccinated**	**276**	**0**	**110**	**70**	**74**	**22**		**20**	
**Vaccine product**									
Combination of mRNA products	6 (2.2)	0 (0.0)	0 (0.0)	0 (0.0)	5 (83.3)	1 (16.7)	NA	14 (8.2)	0.30
Moderna	100 (36.2)	0 (0.0)	40 (40.0)	30 (30.0)	22 (22.0)	8 (8.0)		5 (5.0)	
Pfizer-BioNTech	170 (61.6)	0 (0.0)	70 (41.2)	40 (23.5)	47 (27.6)	13 (7.6)		1 (16.7)	
**Timing of first dose**	**276**								
before current pregnancy	106 (38.4)	0 (0.0)	2 (1.9)	20 (18.9)	62 (58.5)	22 (20.8)	NA	10 (9.4)	0.59
1st trimester	77 (27.9)	0 (0.0)	20 (26.0)	45 (58.4)	12 (15.6)	0 (0.0)		7 (9.1)	
2nd trimester	78 (28.3)	0 (0.0)	73 (93.6)	5 (6.4)	0 (0.0)	0 (0.0)		3 (3.8)	
3rd trimester	15 (5.4)	0 (0.0)	15 (100.0)	0 (0.0)	0 (0.0)	0 (0.0)		0 (0.0)	
**Timing of second dose**	**276**								
before current pregnancy	75 (27.2)	0 (0.0)	0 (0.0)	0 (0.0)	53 (70.7)	22 (29.3)	NA	7 (9.3)	0.60
1st trimester	81 (29.3)	0 (0.0)	8 (9.9)	52 (64.2)	21 (25.9)	0 (0.0)		8 (9.9)	

© 2022 Schrag SJ et al. *JAMA Network Open.*

2nd trimester	89 (32.2)	0 (0.0)	71 (79.8)	18 (20.2)	0 (0.0)	0 (0.0)		5 (5.6)	
3rd trimester	31 (11.2)	0 (0.0)	31 (100.0)	0 (0.0)	0 (0.0)	0 (0.0)		0 (0.0)	
**Timing of third dose**	**96**								
1st trimester	9 (9.4)	0 (0.0)	0 (0.0)	0 (0.0)	2 (22.2)	7 (77.8)	NA	1 (11.1)	0.14
2nd trimester	46 (47.9)	0 (0.0)	0 (0.0)	0 (0.0)	31 (67.4)	15 (32.6)		4 (8.7)	
3rd trimester	41 (42.7)	0 (0.0)	0 (0.0)	0 (0.0)	41 (100.0)	0 (0.0)		3 (7.3)	
**2 Doses**									
**2 doses during current pregnancy**									
No	22 (12.2)	0 (0.0)	2 (9.1)	20 (90.9)	0 (0.0)	0 (0.0)	NA	2 (9.1)	0.14
Yes	158 (87.8)	0 (0.0)	108 (68.4)	50 (31.6)	0 (0.0)	0 (0.0)		10 (6.3)	
**Timing of first dose**	**Timing of 2nd dose**								
before current pregnancy	1st trimester		1	20			NA	2	NA
before current pregnancy	2nd trimester		1	0				0	
1st trimester	1st trimester		7	32				5	
1st trimester	2nd trimester		12	13				2	
1st trimester	3rd trimester		1	0				0	
2nd trimester	2nd trimester		58	5				3	
2nd trimester	3rd trimester		15	0				0	
3rd trimester	3rd trimester		15	0				0	
**3 Doses**									
**3 doses during current pregnancy**									
No	84 (87.5)	0 (0.0)	0 (0.0)	0 (0.0)	62 (73.8)	22 (26.2)	NA	8 (9.5)	0.56
Yes	12 (12.5)	0 (0.0)	0 (0.0)	0 (0.0)	12 (100.0)	0 (0.0)		0 (0.0)	
**Timing of first dose**	**Timing of 2nd dose**	**Timing of 3rd dose**							
before current pregnancy	before current pregnancy	1st trimester	NA		2	7		1	
before current pregnancy	before current pregnancy	2nd trimester			29	15		4	
before current pregnancy	before current pregnancy	3rd trimester			22	0		2	
before current pregnancy	1st trimester	2nd trimester			1	0		0	
before current pregnancy	1st trimester	3rd trimester			8	0		1	
1st trimester	1st trimester	2nd trimester			1	0		0	

© 2022 Schrag SJ et al. *JAMA Network Open.*

1st trimester	1st trimester	3rd trimester		11	0		0	

Abbreviations: ICD-9 = International Classification of Diseases, Ninth Revision; ICD-10 = International Classification of Diseases, Tenth Revision; SMD = standardized mean or proportion difference.

[a]Medical events with an encounter or discharge code consistent with COVID-19–like illness were included, using ICD-9 and ICD-10. Four categories of codes were considered: 1) acute respiratory illness, including respiratory failure, viral or bacterial pneumonia, asthma exacerbation, influenza, and viral illness not otherwise specified; 2) non-respiratory COVID-19–like illness diagnoses including cause-unspecified gastroenteritis, thrombosis, and acute myocarditis; 3) respiratory signs and symptoms consistent with COVID-19–like illness, including hemoptysis, cough, dyspnea, painful respiration, or hypoxemia; 4) signs and symptoms of acute febrile illness. One code in any of the four categories was sufficient for inclusion. Clinician-ordered molecular assays (e.g., real-time reverse transcription–polymerase chain reaction) for SARS-CoV-2 occurring ≤14 days before to <72 hours after the encounter date were included.

[b]Partners contributing data on medical events were in California (estimated start date of Delta predominance June 23, estimated start date of Omicron predominance: December 21), Colorado (estimated start date of Delta predominance June 3, estimated start date of Omicron predominance December 19), Indiana (estimated start date of Delta predominance July 3, estimated start date of Omicron predominance December 26), Minnesota and Wisconsin (estimated start date of Delta predominance July 1, estimated start date of Omicron predominance December 25), New York (estimated start date of Delta predominance June 30, estimated start date of Omicron predominance December 18), Oregon (estimated start date of Delta predominance June 30, estimated start date of Omicron predominance December 24), Texas (estimated start date of Delta predominance September 11, estimated start date of Omicron predominance December 16), Utah (estimated start date of Delta predominance June 1, estimated start date of Omicron predominance December 24), and Washington (estimated star date Delta predominance June 30, estimated start date of Omicron predominance December 24). The study period began in September 2021 for partners located in Texas.

[c] An absolute standardized mean or proportion difference (SMD) ≥0.20 indicates a non-negligible difference in the distribution of characteristics for vaccinated categories versus unvaccinated patients and for positive versus negative test results. When calculating SMDs for differences in characteristics across COVID-19 vaccination status, investigators calculated SMD as the average of the three absolute values of the SMD for unvaccinated versus each vaccination status category individually (unvaccinated versus 2 doses 14-149 days earlier, unvaccinated versus 2-dose >=150 days earlier, and unvaccinated versus 3 mRNA doses ≥7 days earlier). All SMDs are reported as the absolute SMD. Additionally, the average SMD calculation comparing Negative SARS-CoV-2 Test Result and Positive SARS-CoV-2 Test Result was generated by directly calculating the SMD for Negative SARS-CoV-2 Test Result and Positive SARS-CoV-2 Test Result.

[d] Vaccination was defined as having received the listed number of doses of COVID-19 Pfizer-BioNTech BNT162b2 or Moderna mRNA-1273 vaccine ≥14 days (for 2 doses) or ≥7 days (for 3 doses) before the medical event index date, which was the date of respiratory specimen collection associated with the most recent positive or negative SARS-CoV-2 test result before medical event or the admission date if testing only occurred after the admission.

[e]Unknown race/ethnicity includes Asian, Native Hawaiian or other Pacific islander, American Indian or Alaska Native, other not listed, and multiple races.

[f]Chronic respiratory condition was defined as the presence of discharge code for asthma, COPD, or other lung disease using diagnosis codes from the ICD-9 and ICD-10

[g]Chronic non-respiratory condition was defined as the presence of discharge code for heart failure, ischemic heart disease, hypertension, other heart disease, stroke, other cerebrovascular disease, diabetes type I or II, other diabetes, metabolic disease, clinical obesity, clinically underweight, renal disease, liver disease, blood disorder, immunosuppression, organ transplant, cancer, neurological disorder, musculoskeletal disorder, Down Syndrome, and dementia.

© 2022 Schrag SJ et al. *JAMA Network Open.*

eTable 9. Characteristics of Emergency Department and Urgent Care Encounters Among Pregnant People With COVID-19–Like Illness[a] Who Did Not Have a Prior Positive SARS-CoV-2 Infection by COVID-19 mRNA Vaccination Status, VISION Network, 10 States, June 1, 2021 to June 2, 2022[b]

Characteristic	Total, No. (Column %)	No. (Row %)					SMD**	No. (Row %)	SMD**
		mRNA COVID-19 Vaccination Status						Positive SARS-CoV-2 Test Result	
		Unvaccinated	2 doses (14-149 days earlier)	2 doses (≥150 days earlier)	3 doses[¶] (7-119 days earlier)	3 doses[¶] (≥120 days earlier)			
**All ED/UC CLI encounters**	3934	2940	457	175	305	57		828	
**Pregnancy trimester of CLI event**									
1st	1048 (26.6)	940 (89.7)	57 (5.4)	0 (0.0)	51 (4.9)	0 (0.0)	0.82	204 (19.5)	0.07
2nd	1434 (36.5)	1061 (74.0)	198 (13.8)	27 (1.9)	129 (9.0)	19 (1.3)		320 (22.3)	
3rd	1452 (36.9)	939 (64.7)	202 (13.9)	148 (10.2)	125 (8.6)	38 (2.6)		304 (20.9)	
**CLI event associated with delivery**							0.09		0.02
No	3626 (92.2)	2694 (74.3)	433 (11.9)	159 (4.4)	286 (7.9)	54 (1.5)		759 (20.9)	
Yes	308 (7.8)	246 (79.9)	24 (7.8)	16 (5.2)	19 (6.2)	3 (1.0)		69 (22.4)	
**Variant predominance period**									
B.1.617.2 (Delta)	2582 (65.6)	2059 (79.7)	337 (13.1)	112 (4.3)	72 (2.8)	2 (0.1)	0.89	455 (17.6)	0.28
B.1.1.529 (Omicron)	1352 (34.4)	881 (65.2)	120 (8.9)	63 (4.7)	233 (17.2)	55 (4.1)		373 (27.6)	
**Sites**									
Baylor Scott & White Health	81 (2.1)	65 (80.2)	13 (16.0)	1 (1.2)	1 (1.2)	1 (1.2)	0.57	32 (39.5)	0.35
Columbia University	261 (6.6)	185 (70.9)	47 (18.0)	16 (6.1)	12 (4.6)	1 (0.4)		33 (12.6)	
HealthPartners	267 (6.8)	161 (60.3)	37 (13.9)	16 (6.0)	38 (14.2)	15 (5.6)		65 (24.3)	
Intermountain Healthcare	1383 (35.2)	1051 (76.0)	160 (11.6)	58 (4.2)	95 (6.9)	19 (1.4)		220 (15.9)	
Kaiser Permanente Northern California	816 (20.7)	538 (65.9)	128 (15.7)	45 (5.5)	94 (11.5)	11 (1.3)		181 (22.2)	
Kaiser Permanente Northwest	284 (7.2)	202 (71.1)	27 (9.5)	11 (3.9)	38 (13.4)	6 (2.1)		92 (32.4)	
Regenstrief Institute	502 (12.8)	451 (89.8)	26 (5.2)	12 (2.4)	11 (2.2)	2 (0.4)		123 (24.5)	
University of Colorado	340 (8.6)	287 (84.4)	19 (5.6)	16 (4.7)	16 (4.7)	2 (0.6)		82 (24.1)	
**Age (yrs)**									
18-24	1136 (28.9)	982 (86.4)	95 (8.4)	32 (2.8)	22 (1.9)	5 (0.4)	0.54	228 (20.1)	0.04
25-34	2201 (55.9)	1602 (72.8)	260 (11.8)	112 (5.1)	192 (8.7)	35 (1.6)		476 (21.6)	

© 2022 Schrag SJ et al. *JAMA Network Open.*

35-45	597 (15.2)	356 (59.6)	102 (17.1)	31 (5.2)	91 (15.2)	17 (2.8)		124 (20.8)	
**Race/ethnicity**									
Hispanic	1023 (26)	761 (74.4)	146 (14.3)	48 (4.7)	62 (6.1)	6 (0.6)		212 (20.7)	
Non-Hispanic Black	463 (11.8)	403 (87.0)	36 (7.8)	7 (1.5)	15 (3.2)	2 (0.4)	0.49	126 (27.2)	0.13
Non-Hispanic Other[§§]	311 (7.9)	182 (58.5)	46 (14.8)	25 (8.0)	48 (15.4)	10 (3.2)		64 (20.6)	
Non-Hispanic White	2009 (51.1)	1482 (73.8)	222 (11.1)	92 (4.6)	174 (8.7)	39 (1.9)		401 (20.0)	
Unknown	128 (3.3)	112 (87.5)	7 (5.5)	3 (2.3)	6 (4.7)	0 (0.0)		25 (19.5)	
**Chronic respiratory condition[¶¶]**									
No	3651 (92.8)	2720 (74.5)	429 (11.8)	164 (4.5)	284 (7.8)	54 (1.5)	0.05	759 (20.8)	0.05
Yes	283 (7.2)	220 (77.7)	28 (9.9)	11 (3.9)	21 (7.4)	3 (1.1)		69 (24.4)	
**Chronic non respiratory condition[***]**									
No	3622 (92.1)	2700 (74.5)	426 (11.8)	162 (4.5)	283 (7.8)	51 (1.4)	0.05	758 (20.9)	0.02
Yes	312 (7.9)	240 (76.9)	31 (9.9)	13 (4.2)	22 (7.1)	6 (1.9)		70 (22.4)	
**ICU**									
No	3910 (99.4)	2919 (74.7)	457 (11.7)	175 (4.5)	303 (7.7)	56 (1.4)	0.09	820 (21.0)	0.05
Yes	24 (0.6)	21 (87.5)	0 (0.0)	0 (0.0)	2 (8.3)	1 (4.2)		8 (33.3)	
**Immunocompromised status**									
No	3916 (99.5)	2926 (74.7)	455 (11.6)	174 (4.4)	304 (7.8)	57 (1.5)	0.04	825 (21.1)	0.02
Yes	18 (0.5)	14 (77.8)	2 (11.1)	1 (5.6)	1 (5.6)	0 (0.0)		3 (16.7)	
**History of SARSCoV-2 infection**									
No	3934 (100)	2940 (74.7)	457 (11.6)	175 (4.4)	305 (7.8)	57 (1.4)	NA	828 (21.0)	NA
Yes	0 (0)	0 (0)	0 (0)	0 (0)	0 (0)	0 (0)		0 (0)	
**Total vaccinated**	**994**	**0**	**457**	**175**	**305**	**57**		**108**	
**Vaccine product**									
Combination of mRNA products	42 (4.2)	0 (0.0)	0 (0.0)	0 (0.0)	33 (78.6)	9 (21.4)		2 (4.8)	
Moderna	286 (28.8)	0 (0.0)	125 (43.7)	53 (18.5)	89 (31.1)	19 (6.6)	NA	20 (7.0)	0.33
Pfizer-BioNTech	666 (67)	0 (0.0)	332 (49.8)	122 (18.3)	183 (27.5)	29 (4.4)		86 (12.9)	
**Timing of first dose**	**994**								
before current pregnancy	520 (52.3)	0 (0.0)	86 (16.5)	80 (15.4)	297 (57.1)	57 (11.0)		57 (11.0)	
1st trimester	289 (29.1)	0 (0.0)	191 (66.1)	90 (31.1)	8 (2.8)	0 (0.0)		29 (10.0)	
2nd trimester	171 (17.2)	0 (0.0)	166 (97.1)	5 (2.9)	0 (0.0)	0 (0.0)	NA	22 (12.9)	0.20
3rd trimester	14 (1.4)	0 (0.0)	14 (100.0)	0 (0.0)	0 (0.0)	0 (0.0)		0 (0.0)	
**Timing of second dose**	**994**								

© 2022 Schrag SJ et al. *JAMA Network Open.*

before current pregnancy	348 (35)	0 (0.0)	0 (0.0)	0 (0.0)	291 (83.6)	57 (16.4)	NA	33 (9.5)	0.26
1st trimester	401 (40.3)	0 (0.0)	229 (57.1)	158 (39.4)	14 (3.5)	0 (0.0)		50 (12.5)	
2nd trimester	205 (20.6)	0 (0.0)	188 (91.7)	17 (8.3)	0 (0.0)	0 (0.0)		24 (11.7)	
3rd trimester	40 (4)	0 (0.0)	40 (100.0)	0 (0.0)	0 (0.0)	0 (0.0)		1 (2.5)	
**Timing of third dose**	**362**								
1st trimester	155 (42.8)	0 (0.0)	0 (0.0)	0 (0.0)	115 (74.2)	40 (25.8)	NA	16 (10.3)	0.10
2nd trimester	165 (45.6)	0 (0.0)	0 (0.0)	0 (0.0)	148 (89.7)	17 (10.3)		14 (8.5)	
3rd trimester	42 (11.6)	0 (0.0)	0 (0.0)	0 (0.0)	42 (100.0)	0 (0.0)		4 (9.5)	
**2 Doses**									
**2 doses during current pregnancy**									
No	166 (26.3)	0 (0.0)	86 (51.8)	80 (48.2)	0 (0.0)	0 (0.0)	NA	23 (13.9)	0.12
Yes	466 (73.7)	0 (0.0)	371 (79.6)	95 (20.4)	0 (0.0)	0 (0.0)		51 (10.9)	
**Timing of first dose**	**Timing of 2nd dose**								
before current pregnancy	1st trimester		85	80	NA		NA	23	NA
before current pregnancy	2nd trimester		1	0				0	
1st trimester	1st trimester		144	78				26	
1st trimester	2nd trimester		45	12				3	
1st trimester	3rd trimester		2	0				0	
2nd trimester	2nd trimester		142	5				21	
2nd trimester	3rd trimester		24	0				1	
3rd trimester	3rd trimester		14	0				0	
**3 Doses**									
**3 doses during current pregnancy**									
No	354 (97.8)	0 (0.0)	0 (0.0)	0 (0.0)	297 (83.9)	57 (16.1)	NA	34 (9.6)	0.22
Yes	8 (2.2)	0 (0.0)	0 (0.0)	0 (0.0)	8 (100.0)	0 (0.0)		0 (0.0)	
**Timing of first dose**	**Timing of 2nd dose**	**Timing of 3rd dose**							
before current pregnancy	before current pregnancy	1st trimester	NA		115	40	NA	16	NA
before current pregnancy	before current pregnancy	2nd trimester			147	17		14	
before current pregnancy	before current pregnancy	3rd trimester			29	0		0	

© 2022 Schrag SJ et al. *JAMA Network Open.*

before current pregnancy	1st trimester	2nd trimester		1	0		0	
before current pregnancy	1st trimester	3rd trimester		5	0		3	
1st trimester	1st trimester	3rd trimester		8	0		1	

Abbreviations: ICD-9 = International Classification of Diseases, Ninth Revision; ICD-10 = International Classification of Diseases, Tenth Revision; SMD = standardized mean or proportion difference.

[a]Medical events with an encounter or discharge code consistent with COVID-19–like illness were included, using ICD-9 and ICD-10. Four categories of codes were considered: 1) acute respiratory illness, including respiratory failure, viral or bacterial pneumonia, asthma exacerbation, influenza, and viral illness not otherwise specified; 2) non-respiratory COVID-19–like illness diagnoses including cause-unspecified gastroenteritis, thrombosis, and acute myocarditis; 3) respiratory signs and symptoms consistent with COVID-19–like illness, including hemoptysis, cough, dyspnea, painful respiration, or hypoxemia; 4) signs and symptoms of acute febrile illness. One code in any of the four categories was sufficient for inclusion. Clinician-ordered molecular assays (e.g., real-time reverse transcription–polymerase chain reaction) for SARS-CoV-2 occurring ≤14 days before to <72 hours after the encounter date were included.

[b]Partners contributing data on medical events were in California (estimated start date of Delta predominance June 23, estimated start date of Omicron predominance: December 21), Colorado (estimated start date of Delta predominance June 3, estimated start date of Omicron predominance December 19), Indiana (estimated start date of Delta predominance July 3, estimated start date of Omicron predominance December 26), Minnesota and Wisconsin (estimated start date of Delta predominance July 1, estimated start date of Omicron predominance December 25), New York (estimated start date of Delta predominance June 30, estimated start date of Omicron predominance December 18), Oregon (estimated start date of Delta predominance June 30, estimated start date of Omicron predominance December 24), Texas (estimated start date of Delta predominance September 11, estimated start date of Omicron predominance December 16), Utah (estimated start date of Delta predominance June 1, estimated start date of Omicron predominance December 24), and Washington (estimated star date Delta predominance June 30, estimated start date of Omicron predominance December 24). The study period began in September 2021 for partners located in Texas.

[c] An absolute standardized mean or proportion difference (SMD) ≥0.20 indicates a non-negligible difference in the distribution of characteristics for vaccinated categories versus unvaccinated patients and for positive versus negative test results. When calculating SMDs for differences in characteristics across COVID-19 vaccination status, investigators calculated SMD as the average of the three absolute values of the SMD for unvaccinated versus each vaccination status category individually (unvaccinated versus 2 doses 14-149 days earlier, unvaccinated versus 2-dose >=150 days earlier, and unvaccinated versus 3 mRNA doses ≥7 days earlier). All SMDs are reported as the absolute SMD. Additionally, the average SMD calculation comparing Negative SARS-CoV-2 Test Result and Positive SARS-CoV-2 Test Result was generated by directly calculating the SMD for Negative SARS-CoV-2 Test Result and Positive SARS-CoV-2 Test Result.

[d] Vaccination was defined as having received the listed number of doses of COVID-19 Pfizer-BioNTech BNT162b2 or Moderna mRNA-1273 vaccine ≥14 days (for 2 doses) or ≥7 days (for 3 doses) before the medical event index date, which was the date of respiratory specimen collection associated with the most recent positive or negative SARS-CoV-2 test result before medical event or the admission date if testing only occurred after the admission.

[e]Unknown race/ethnicity includes Asian, Native Hawaiian or other Pacific islander, American Indian or Alaska Native, other not listed, and multiple races.

[f]Chronic respiratory condition was defined as the presence of discharge code for asthma, COPD, or other lung disease using diagnosis codes from the ICD-9 and ICD-10

[g]Chronic non-respiratory condition was defined as the presence of discharge code for heart failure, ischemic heart disease, hypertension, other heart disease, stroke, other cerebrovascular disease, diabetes type I or II, other diabetes, metabolic disease, clinical obesity, clinically underweight, renal disease, liver disease, blood disorder, immunosuppression, organ transplant, cancer, neurological disorder, musculoskeletal disorder, Down Syndrome, and dementia.

© 2022 Schrag SJ et al. *JAMA Network Open.*

eFigure1. Flow Figure for Hospitalizations and ED/UC Visits Among Pregnant People (Unvaccinated or With 1 Vaccine Dose Received During Pregnancy)[a]

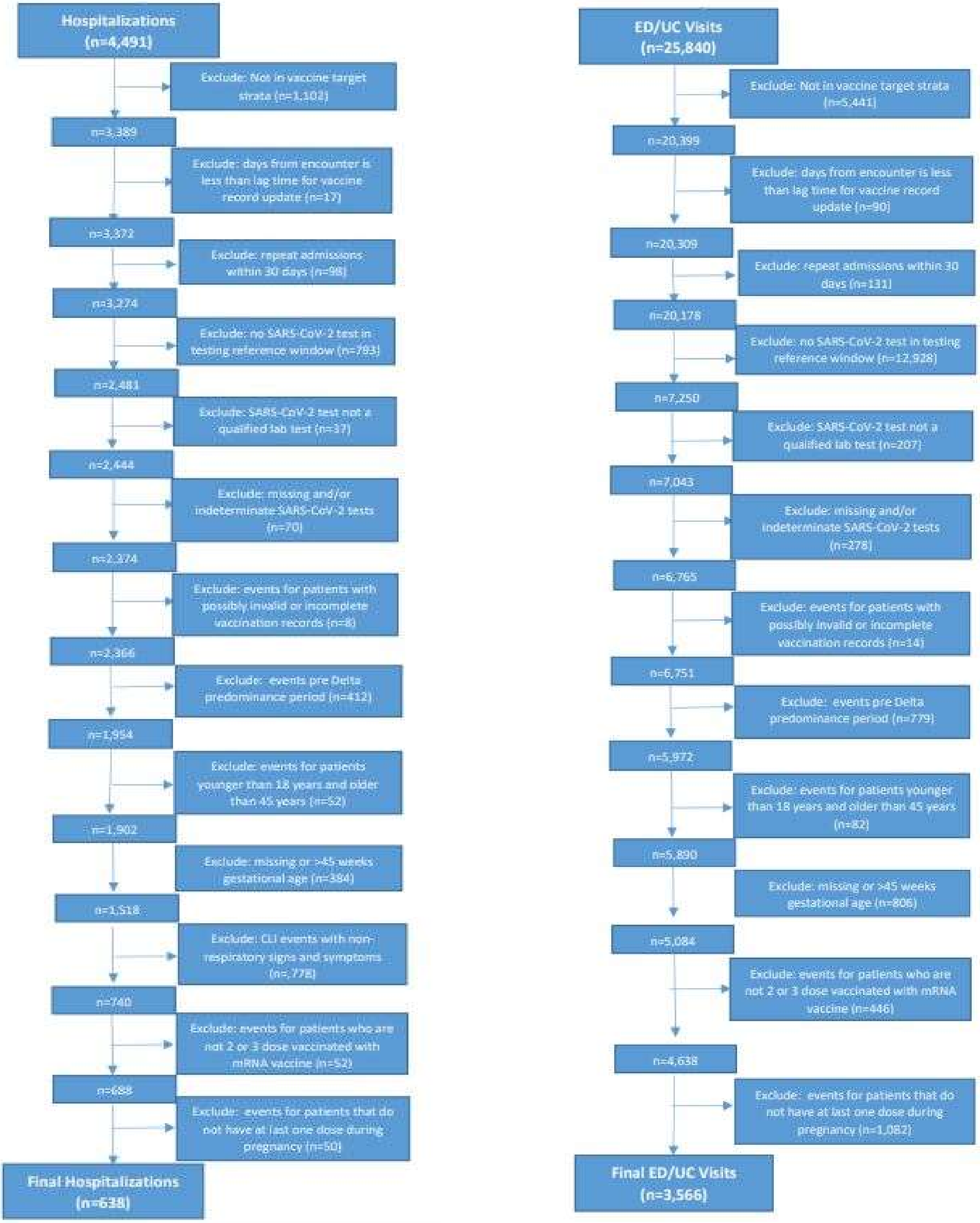

© 2022 Schrag SJ et al. *JAMA Network Open.*

eFigure 2. mRNA COVID-19 Vaccine Effectiveness Against Laboratory-Confirmed COVID-19–Associated Emergency Department and Urgent Care Encounter Among Pregnant People Regardless of Pregnancy Status at Time of Vaccination, VISION Network, 10 States, June 1, 2021, to June 2, 2022

Vaccination Status	Cases	(Col %)	Controls	(Col%)	Total	(SARS CoV-2 + %)	VE % (CI)
Vaccination Either Before or During Pregnancy							
Omicron Time Period							
Unvaccinated (Referent)	325	(50.9)	773	(40.2)	1098	(29.6)	
2-dose mRNA vaccinated >=14 days prior	262	(41.1)	707	(36.8)	969	(27)	20 (-1 to 37)
2-dose mRNA vaccinated 14-149 days prior	70	(11.0)	169	(8.8)	239	(29.3)	39 (13 to 58)
2-dose mRNA vaccinated >= 150 days prior	192	(30.1)	538	(28.0)	730	(26.3)	11 (-16 to 32)
3-dose mRNA vaccinated >= 7 days prior	51	(8.0)	443	(23.0)	494	(10.3)	65 (48 to 77)
3-dose mRNA vaccinated 7-119 days prior	26	(4.1)	316	(16.4)	342	(7.6)	81 (68 to 89)
3-dose mRNA vaccinated >= 120 days prior	25	(3.9)	127	(6.6)	152	(16.4)	-26 (-123 to 29)
Delta Time Period							
Unvaccinated (Referent)	443	(88.1)	1839	(58.7)	2282	(19.4)	
2-dose mRNA vaccinated >=14 days prior	55	(10.9)	1204	(38.4)	1259	(4.4)	83 (75 to 88)
2-dose mRNA vaccinated 14-149 days prior	22	(4.4)	601	(19.2)	623	(3.5)	87 (78 to 92)
2-dose mRNA vaccinated >= 150 days prior	33	(6.6)	603	(19.3)	636	(5.2)	78 (64 to 87)
3-dose mRNA vaccinated >= 7 days prior	5	(1.0)	89	(2.8)	94	(5.3)	83 (57 to 94)
3-dose mRNA vaccinated 7-119 days prior	5	(1.0)	86	(2.7)	91	(5.5)	82 (53 to 93)
3-dose mRNA vaccinated >= 120 days prior	0	(0.0)	3	(0.1)	3	(0)	

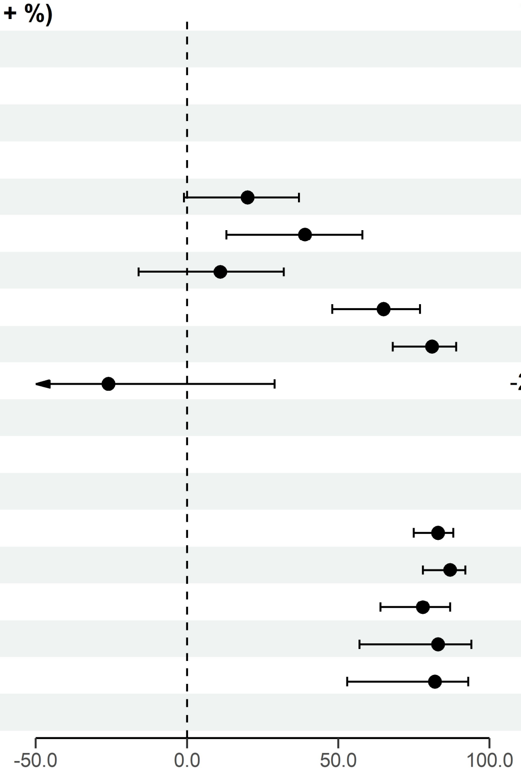

VE could not be calculated for Delta Period, 3-dose mRNA vaccinated ≥120 days because the stratum included fewer than 20 encounters with prior vaccination.

© 2022 Schrag SJ et al. *JAMA Network Open.*

eFigure 3. mRNA COVID-19 Vaccine Effectiveness Against Laboratory-Confirmed COVID-19–Associated Hospitalizations Among Pregnant People Regardless of Pregnancy Status at Time of Vaccination, VISION Network, 10 states, June 1, 2021, to June 2, 2022

Vaccination Status	Cases	(Col %)	Controls	(Col%)	Total	(SARS CoV-2 + %)	VE % (CI)
Vaccination Either Before or During Pregnancy							
Omicron Time Period							
Unvaccinated (Referent)	60	(64.5)	112	(40.9)	172	(34.9)	
2-dose mRNA vaccinated >=14 days prior	24	(25.8)	83	(30.3)	107	(22.4)	58 (12 to 80)
2-dose mRNA vaccinated 14-149 days prior	4	(4.3)	17	(6.2)	21	(19)	82 (41 to 95)
2-dose mRNA vaccinated >= 150 days prior	20	(21.5)	66	(24.1)	86	(23.3)	40 (-44 to 75)
3-dose mRNA vaccinated >= 7 days prior	9	(9.7)	79	(28.8)	88	(10.2)	85 (53 to 95)
3-dose mRNA vaccinated 7-119 days prior	4	(4.3)	57	(20.8)	61	(6.6)	94 (66 to 99)
3-dose mRNA vaccinated >= 120 days prior	5	(5.4)	22	(8.0)	27	(18.5)	-61 (-601 to 63)
Delta Time Period							
Unvaccinated (Referent)	253	(98.1)	245	(54.7)	498	(50.8)	
2-dose mRNA vaccinated >=14 days prior	4	(1.6)	181	(40.4)	185	(2.2)	99 (97 to 100)
2-dose mRNA vaccinated 14-149 days prior	1	(0.4)	101	(22.5)	102	(1)	99 (96 to 100)
2-dose mRNA vaccinated >= 150 days prior	3	(1.2)	80	(17.9)	83	(3.6)	98 (92 to 99)
3-dose mRNA vaccinated >= 7 days prior	1	(0.4)	22	(4.9)	23	(4.3)	98 (80 to 100)
3-dose mRNA vaccinated 7-119 days prior	1	(0.4)	22	(4.9)	23	(4.3)	98 (80 to 100)
3-dose mRNA vaccinated >= 120 days prior	0	(0.0)	0	(0.0)	0	(0)	

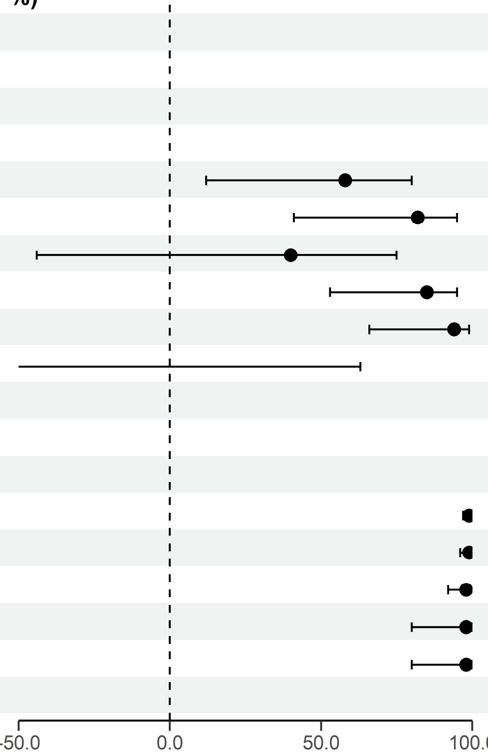

VE could not be calculated for Delta Period, 3-dose mRNA vaccinated ≥120 days because the stratum included fewer than 20 encounters with prior vaccination.

© 2022 Schrag SJ et al. *JAMA Network Open.*

eFigure 4. mRNA COVID-19 Vaccine Effectiveness Against Laboratory-Confirmed COVID-19–Associated Emergency Department and Urgent Care Encounter Among Pregnant People Who Did Not Have a Prior Positive SARS-CoV-2 Infection, VISION Network, 10 States, June 1, 2021, to June 2, 2022

Vaccination Status	Cases	(Col %)	Controls	(Col%)	Total	(SARS CoV-2 + %)	VE % (CI)
No History of Prior SARS CoV-2 Infection							
Omicron Time Period							
Unvaccinated (Referent)	285	(76.4)	596	(60.9)	881	(32.3)	
2-dose mRNA vaccinated >=14 days prior	58	(15.5)	125	(12.8)	183	(31.7)	15 (-26 to 43)
2-dose mRNA vaccinated 14-149 days prior	44	(11.8)	76	(7.8)	120	(36.7)	-7 (-69 to 33)
2-dose mRNA vaccinated >= 150 days prior	14	(3.8)	49	(5.0)	63	(22.2)	54 (7 to 77)
3-dose mRNA vaccinated >= 7 days prior	30	(8.0)	258	(26.4)	288	(10.4)	67 (43 to 80)
3-dose mRNA vaccinated 7-119 days prior	21	(5.6)	212	(21.7)	233	(9)	79 (60 to 89)
3-dose mRNA vaccinated >= 120 days prior	9	(2.4)	46	(4.7)	55	(16.4)	-163 (-540 to -8)
Delta Time Period							
Unvaccinated (Referent)	435	(95.6)	1624	(76.4)	2059	(21.1)	
2-dose mRNA vaccinated >=14 days prior	16	(3.5)	433	(20.4)	449	(3.6)	84 (69 to 91)
2-dose mRNA vaccinated 14-149 days prior	12	(2.6)	325	(15.3)	337	(3.6)	86 (71 to 93)
2-dose mRNA vaccinated >= 150 days prior	4	(0.9)	108	(5.1)	112	(3.6)	74 (4 to 93)
3-dose mRNA vaccinated >= 7 days prior	4	(0.9)	70	(3.3)	74	(5.4)	81 (35 to 95)
3-dose mRNA vaccinated 7-119 days prior	4	(0.9)	68	(3.2)	72	(5.6)	81 (33 to 94)
3-dose mRNA vaccinated >= 120 days prior	0	(0.0)	2	(0.1)	2	(0)	

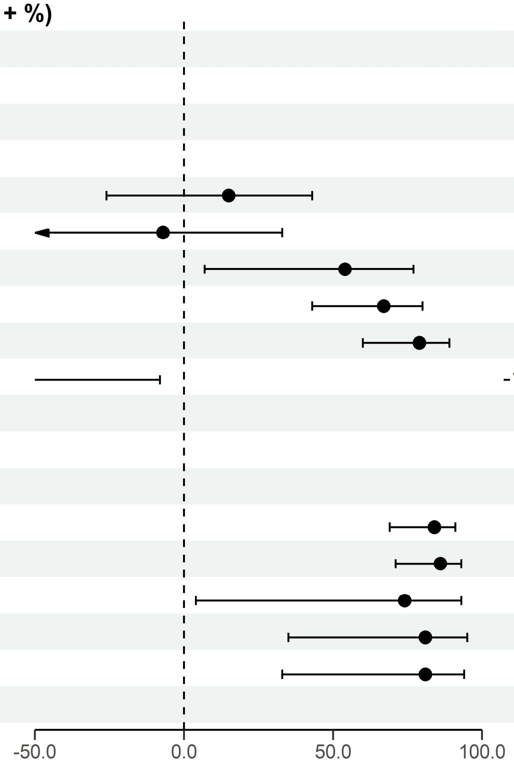

VE could not be calculated for Delta Period, 3-dose mRNA vaccinated ≥120 days because the stratum included fewer than 20 encounters with prior vaccination.

© 2022 Schrag SJ et al. *JAMA Network Open.*

eFigure 5. mRNA COVID-19 Vaccine Effectiveness Against Laboratory-Confirmed COVID-19–Associated Hospitalizations Among Pregnant People Who Did Not Have a Prior Positive SARS-CoV-2 Infection, VISION Network, 10 States, June 1, 2021, to June 2, 2022

Vaccination Status	Cases	(Col %)	Controls	(Col%)	Total	(SARS CoV-2 + %)	VE % (CI)
No History of Prior SARS CoV-2 Infection							
Omicron Time Period							
Unvaccinated (Referent)	59	(79.7)	84	(47.7)	143	(41.3)	
2-dose mRNA vaccinated >=14 days prior	8	(10.8)	24	(13.6)	32	(25)	75 (7 to 93)
2-dose mRNA vaccinated 14-149 days prior	4	(5.4)	12	(6.8)	16	(25)	80 (5 to 96)
2-dose mRNA vaccinated >= 150 days prior	4	(5.4)	12	(6.8)	16	(25)	69 (-142 to 96)
3-dose mRNA vaccinated >= 7 days prior	7	(9.5)	68	(38.6)	75	(9.3)	82 (45 to 94)
3-dose mRNA vaccinated 7-119 days prior	4	(5.4)	49	(27.8)	53	(7.5)	90 (61 to 97)
3-dose mRNA vaccinated >= 120 days prior	3	(4.1)	19	(10.8)	22	(13.6)	-65 (-1817 to 86)
Delta Time Period							
Unvaccinated (Referent)	250	(98.0)	219	(57.2)	469	(53.3)	
2-dose mRNA vaccinated >=14 days prior	4	(1.6)	144	(37.6)	148	(2.7)	98 (96 to 99)
2-dose mRNA vaccinated 14-149 days prior	1	(0.4)	93	(24.3)	94	(1.1)	99 (96 to 100)
2-dose mRNA vaccinated >= 150 days prior	3	(1.2)	51	(13.3)	54	(5.6)	96 (87 to 99)
3-dose mRNA vaccinated >= 7 days prior	1	(0.4)	20	(5.2)	21	(4.8)	97 (74 to 100)
3-dose mRNA vaccinated 7-119 days prior	1	(0.4)	20	(5.2)	21	(4.8)	97 (74 to 100)
3-dose mRNA vaccinated >= 120 days prior	0	(0.0)	0	(0.0)	0	(0)	

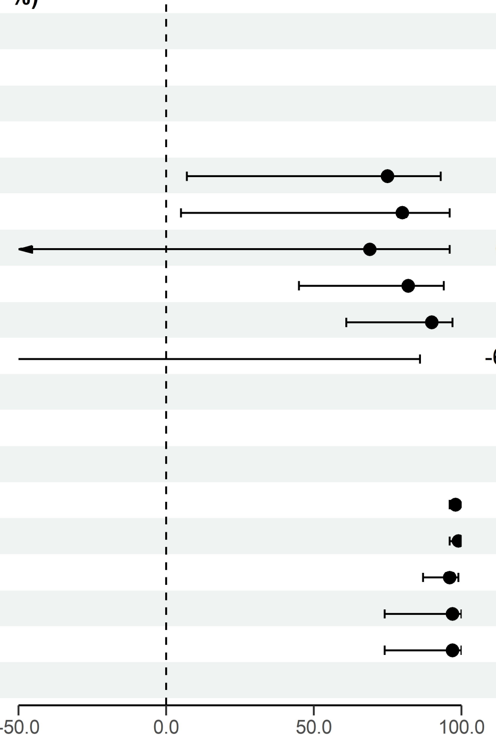

VE could not be calculated for Delta Period, 3-dose mRNA vaccinated ≥120 days because the stratum included fewer than 20 encounters with prior vaccination.

© 2022 Schrag SJ et al. *JAMA Network Open.*
